# Complex precursor structures of cytolytic cupiennins identified in spider venom gland transcriptomes

**DOI:** 10.1038/s41598-021-83624-z

**Published:** 2021-02-17

**Authors:** Lucia Kuhn-Nentwig

**Affiliations:** Institute of Ecology and Evolution, University of Bern, Baltzerstrasse 6, 3012 Bern, Switzerland

**Keywords:** Post-translational modifications, Peptides, Cell biology, Molecular biology, Transcriptomics

## Abstract

Analysis of spider venom gland transcriptomes focuses on the identification of possible neurotoxins, proteins and enzymes. Here, the first comprehensive transcriptome analysis of cupiennins, small linear cationic peptides, also known as cytolytic or antimicrobial peptides, is reported from the venom gland transcriptome of *Cupiennius salei* by 454- and Illumina 3000 sequencing. Four transcript families with complex precursor structures are responsible for the expression of 179 linear peptides. Within the transcript families, after an anionic propeptide, cationic linear peptides are separated by anionic linkers, which are transcript family specific. The C-terminus of the transcript families is characterized by a linear peptide or truncated linkers with unknown function. A new identified posttranslational processing mechanism explains the presence of the two-chain CsTx-16 family in the venom. The high diversity of linear peptides in the venom of a spider and this unique synthesis process is at least genus specific as verified with *Cupiennius getazi*.

## Introduction

Spider can be traced back to around 315 million years^1^. They populate nearly all ecosystems and are with more than 48,770 species^2^ the most species-rich terrestrial invertebrate group after insects. Evolution during such a long time generated species that mainly use venom as generalized predators, besides a few specialized groups^3^. Spider venom has to fulfill three tasks, (1) immediate paralysis of prey, (2) fast acting in lowest doses, and (3) defense against aggressors. Spider venom is composed of a huge diversity of compounds, which differs in composition among spider families^4^. Besides low molecular mass compounds, proteins and enzymes, peptides are the key players in the envenomation process of most spiders, but for humans they are also a rich basis for agricultural and medical applications^5^. Most knowledge of such peptides refers to classical disulfide-rich peptide neurotoxins^6,7^. Small linear peptides (LPs) in animal venoms are known from many arthropods^8^ and a very limited number of araneomorph spiders^9^. So far, LPs were only identified in spiders of the RTA-clade, namely zodariids^10^, lycosids^11,12^, oxyopids^13^, and *Cupiennius salei*^14,15^.

Analyses of spider venom gland transcriptomes opened the possibility to obtain more information on peptide precursors and their maturing. Classical disulfide containing neurotoxins are composed of a signal peptide, followed by a propeptide and the mature peptide, which is posttranslationally modified (simple precursor structure). Kozlov and Grishin^16^ identified C-terminally of the propeptide a conserved motif of four amino acid residues, as recognition site for a specific venom protease^17^, which is the key for peptide maturation. This motif is named Processing Quadruplet Motif (PQM) and it is characterized by an Arg residue at position-1, and at least one Glu at position-2 to -4. Further, infrequently used dibasic cleavage motifs are RR, KR, RK, and KK^16^. The former Grishin group^10^ identified different precursors of cytolytic LPs (latarcins) in zodariids as simple, binary and complex precursors. In the case of complex precursors, they identified N-terminally of the PQM containing peptide an inverted PQM (iPQM) of four amino acid residues, starting with Arg, and with Glu at least in one of the next three positions^9^. So far, first insights into the LPs precursor and maturing process of seven latarcin families, comprising twelve latarcins from *Lachesana tarabaevi,* were published^9^. They showed that most of the latarcins are encoded as simple precursors, namely one transcript results in one peptide. Beside this, two latarcins are encoded in complex transcripts, with four to five repetitive elements before the C-terminally occurring latarcins 4a or 4b. Unique is the identification of cyto-insectotoxins by Vassilevski and coworkers in this venom, which are the longest so far identified linear, cationic peptides with lengths between 62 and 79 amino acid residues, acting strong insecticidal^18^.

Investigations of small linear peptides (LPs) in the venom of *C. salei* started nearly 20 years ago on proteomic site, using classical chromatographic methods as gel filtration, ion exchange- and RP-HPLC^14^. The amino acid sequence of purified peptides were first elucidated by traditional Edman degradation and later with tandem mass spectrometry (MALDI–TOF–MS and ESI–MS)^15^. As a result, 26 small LPs were identified and aside from that, several truncated variants of such peptides. Surprisingly, Cupiennin 1a is in the millimolar range (1.2 mM) present in the venom, comparable to the concentration of the most abundant neurotoxin CsTx-1 as published earlier^14^. Hindered through high sequence identities, a huge diversity of truncated peptide variants in the venom, and nearly identical elution times in RP-HPLC, we started transcriptome analysis of the venom glands of *C. salei* by 454-sequencing (454-seq)^19,20^ and later by NGS^17,21^. Strikingly, 454-transcriptome analysis disclosed that LPs are with 24% of all annotated contigs considerably more expressed than disulfide containing neurotoxins (15%)^20^. Due to the shorter read length obtained by NGS^7^, the high sequence similarity, and the high number of different linear, mainly cationic peptides, 454-sequencing transcriptomic data was preferably used for the analysis of the complex transcript structures. NGS data was used to supplement and to clarify critical data.

Optimization is the key function for venom usage^22^, venom composition^20^ and synergistic interactions of its components^23–25^, as described for *C. salei*. Which optimization strategy is behind the production of numerous small, cationic peptides? How complex are the LP precursor structures in *C. salei* in terms of simple, binary or complex precursors? Are new posttranslational processing strategies detectable and are these results applicable to other spiders? Here, the first holistic description of the nature of small, cationic LPs within a spider’s venom strategy is outlined.

## Results

**Figure 1:**
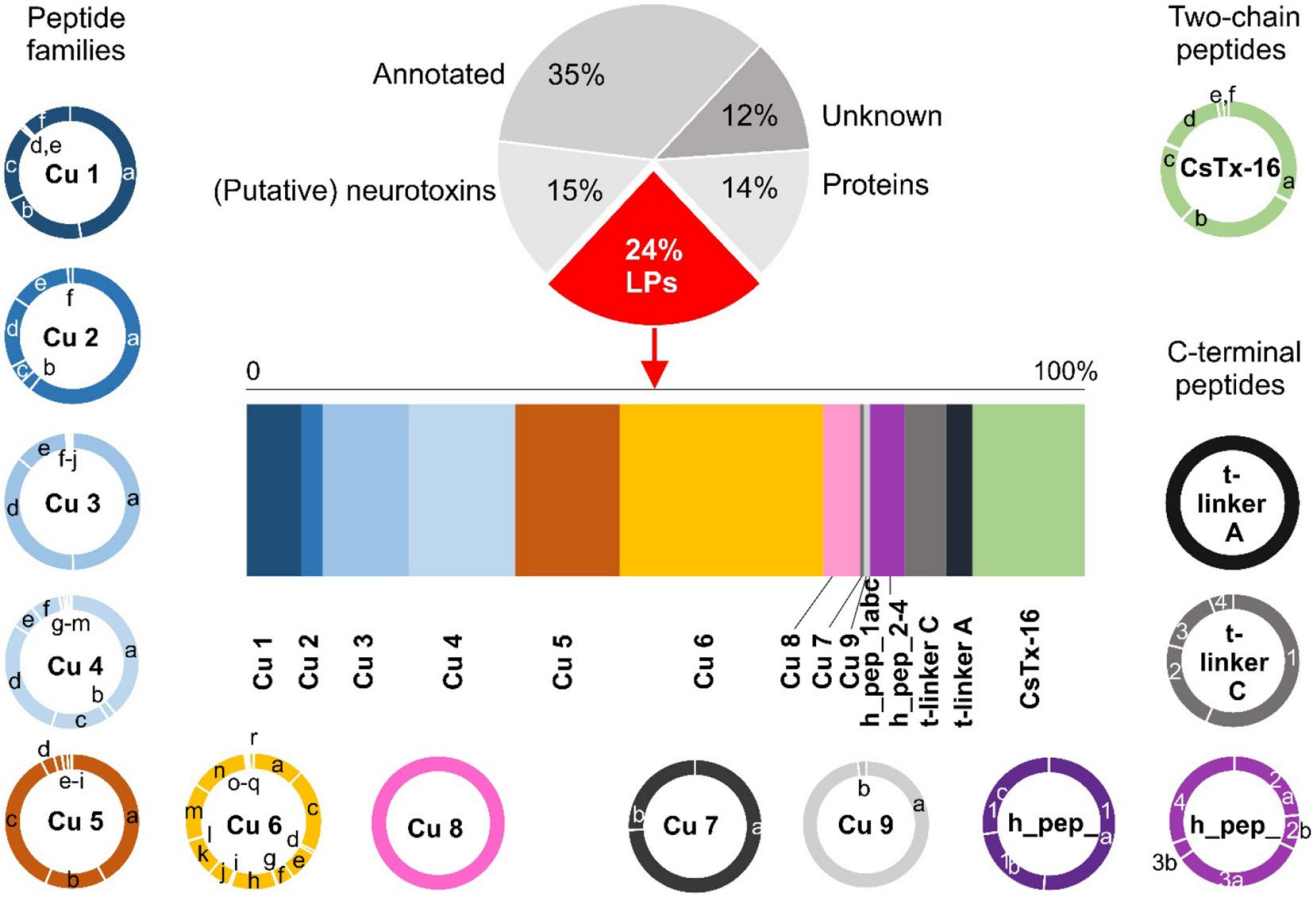
Transcriptome analysis of the venom glands of *C. salei*. The upper pie chart shows the distribution of all identified contigs referring to peptides and proteins^20^. Below this, the percentage distribution (reads) of all identified LPs is presented. The distribution and quantity of related peptides within the different peptide families are also illustrated in pie charts, in identical colors, corresponding to their percentage presence in the transcriptome. CsTx-16 is counted as two-chain peptide.

Transcriptome analysis of *Cupiennius salei* venom glands discloses the existence of a very high number of small, in majority Cys free cationic peptides, which is unique among spiders^20^. About 24% of all transcripts of *C. salei* refer to such linear peptides (LPs) and they can be grouped into nine different cupiennin families (Cu 1–9), one CsTx-16 family, and four hypothetical peptide families (h_pep_1-4) (Fig. 1).

### Transcript compositions

**Figure 2:**
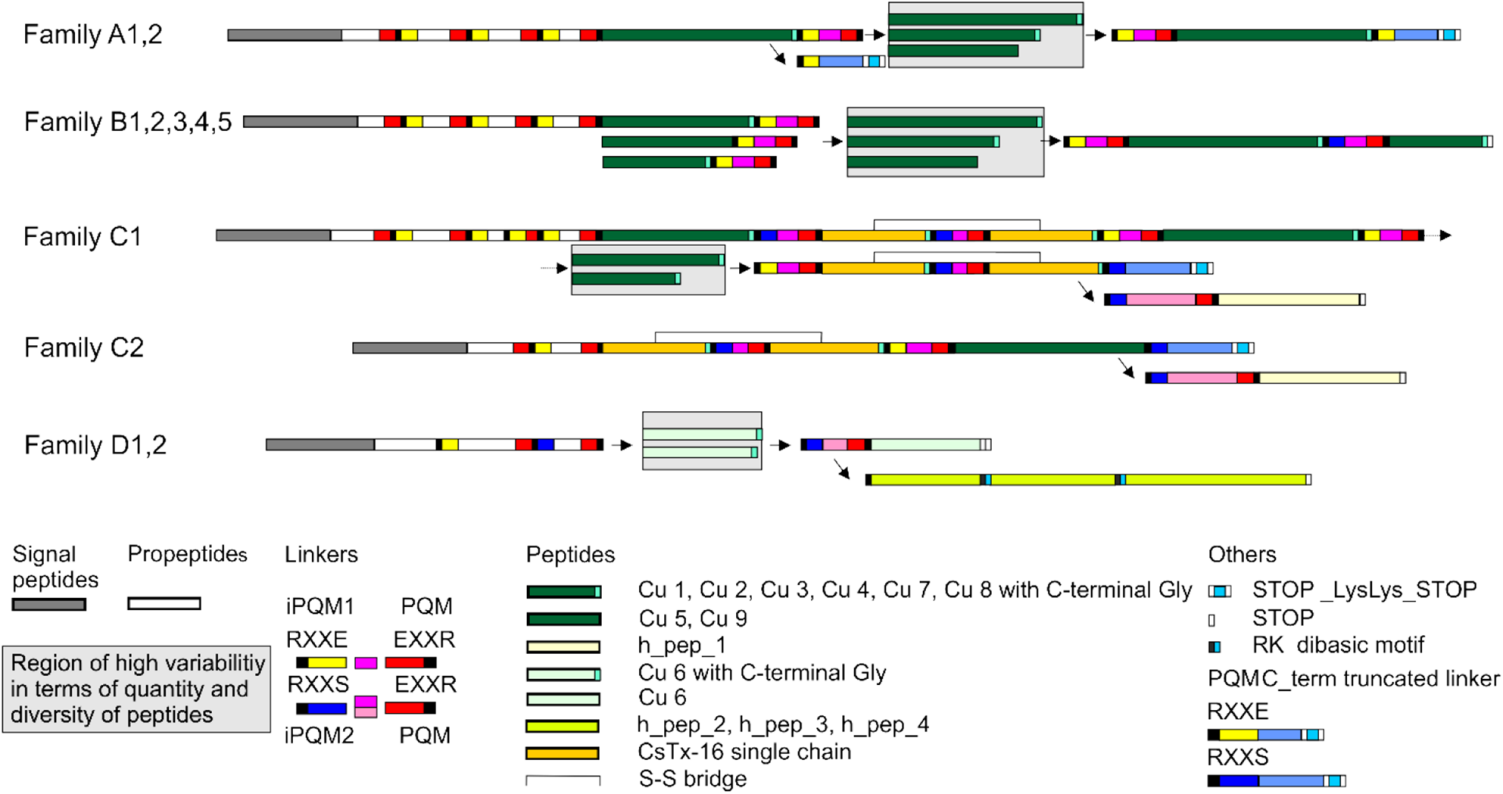
Overview on the construction of transcript families (**A**–**D**), encoding different LPs. Signal peptides, propeptides, and linkers, which are connecting different LPs, are illustrated as explained by symbols in the lower part of the figure. Likewise, differences in iPQM and PQMs structures, as well as in the C-terminus of the transcripts are colored corresponding to the symbol explanations.

On the first view, four main transcript families (A–D) encode the different LPs in more or less complex precursor structures enclosing more than ten different LPs, all separated by linkers and ending in two different C-terminal structure families. Minor differences, however, allow to distinguish two variants for the A, C, D families, and five variants for the B family (Fig. 2, S1A-E Fig).

### Signal peptides and propeptides

In general, transcripts encoding LPs are composed of short signal peptides followed by different propeptides and small LPs, which are separated by short, anionic amino acid residues. Such elements are defined as spacers or linkers and are characterized N-terminally by an inverted processing quadruplet motif (iPQM) and C-terminally by a processing quadruplet motif (PQM). Signal peptides are composed of 21 amino acid residues (transcripts A, B, C) or 20 amino acid residues (transcript D). The lengths of the anionic propeptides (theoretical pIs 3.85–4.33) vary between 25 and 42–49 amino acid residues and more than one third of them are Glu and Asp. The propeptides exhibit α-helical structures (28–70%) and dependent on their lengths, three to four PQMs and two to three iPQMs. Additionally, the C-terminal parts of the propeptides are used one to four times as linkers between the peptides within the corresponding transcript (transcripts A, B, C). Interestingly, on protein level, transcript B and C1 exhibit identical signal peptides and differ from the signal peptide of transcript C2 in only one amino acid residue and from transcript A in three amino acid residues. Propeptides of all four transcript families exhibit major differences in amino acid composition and length (S1A-E Fig).

### Linkers

All LPs from *C. salei* are separated by linkers within the corresponding transcript. For data analysis, a linker is defined as a short amino acid sequence starting and ending with Arg and exhibiting both, iPQM and PQM. However, posttranslational processing by proteases will occur always after Arg and the remaining C-terminal Arg of the foregoing peptide and of the linker will be removed by a so far unknown Arg-C proteinase^17^.

Linkers are mainly transcript family specific, even though the linker RTENEIDEEDER belongs to the C-terminal part of propeptides A2 as well as B2 and links different peptides: Cu 1a with Cu 4c (transcript A) as well as Cu 8 with Cu 3d, Cu 3d with Cu 4a, Cu 4e with Cu 4a, and Cu 4f with Cu 1a (transcript B). All linkers within transcript A (n = 18) and B (n = 10) are composed of 12 amino acid residues and exhibit at least one Glu in the iPQM (iPQM1 variant) and in the PQM. Characteristic are molecular masses around 1529 Da, and a mean anionic pI of 4.08, which is caused by five to seven Glu/Asp residues per linker.

Nearly half of the linkers of transcript C1 and C2 are composed of 11 amino acid residues and the N-terminal iPQM possesses no Glu residue but a Ser residue (iPQM2 variant). Such linkers are identified only before and between CsTx-16 single peptide chains (n = 3). The mean molecular mass is 1284 Da and these linkers exhibit a higher pI (4.44) due to less Glu/Asp in its sequences. Remarkably, in transcript C2 one linker, which is composed of 13 amino residues, was identified and also confirmed by NGS. Also, all linkers of transcript D peptides exhibit 13 amino acid residues, and show structurally only few differences. Six main linkers connect the most diverse members of the LP family Cu 6. Comparable to some transcript C linkers, the N-terminal Glu is replaced by a Ser and a Thr residue.

In total, we identified a minimum of 43 different linker sequences by 454-seq, responsible for the arrangement of different LPs in different transcripts (Table 1).

**Table 1:**
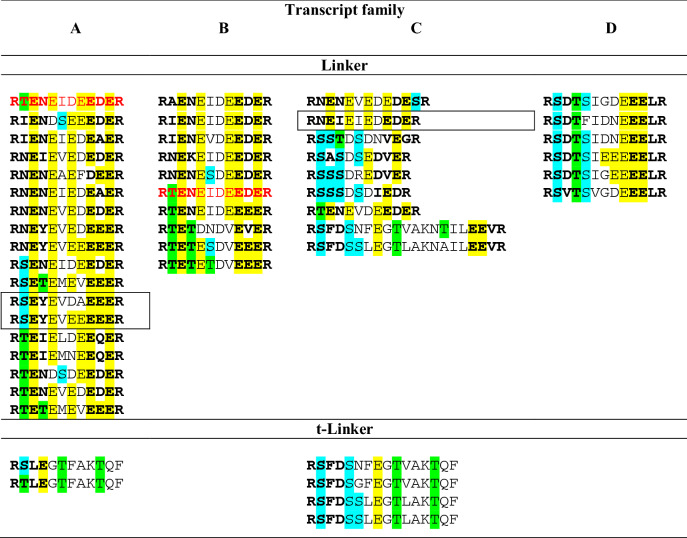
Overview on main transcript specific LPs linkers and t-linkers identified in the venom gland transcriptome of *C. salei* by 454-sequencing. Marked in bold are iPQM and PQM. E, T, and S are marked in different colors for a better visualization. Identical linkers in different transcripts are colored in red. Linkers not verified by NGS are boxed in black.

Besides these linkers, C-terminal truncated linkers (t-linkers) were identified in transcript A and C, which exhibit iPQM1 and iPQM2 structures, no C-terminal PQM and are followed by a stop signal, two Lys residues and again a stop signal (S1A-E Fig).

### Transcript families

**Figure 3:**
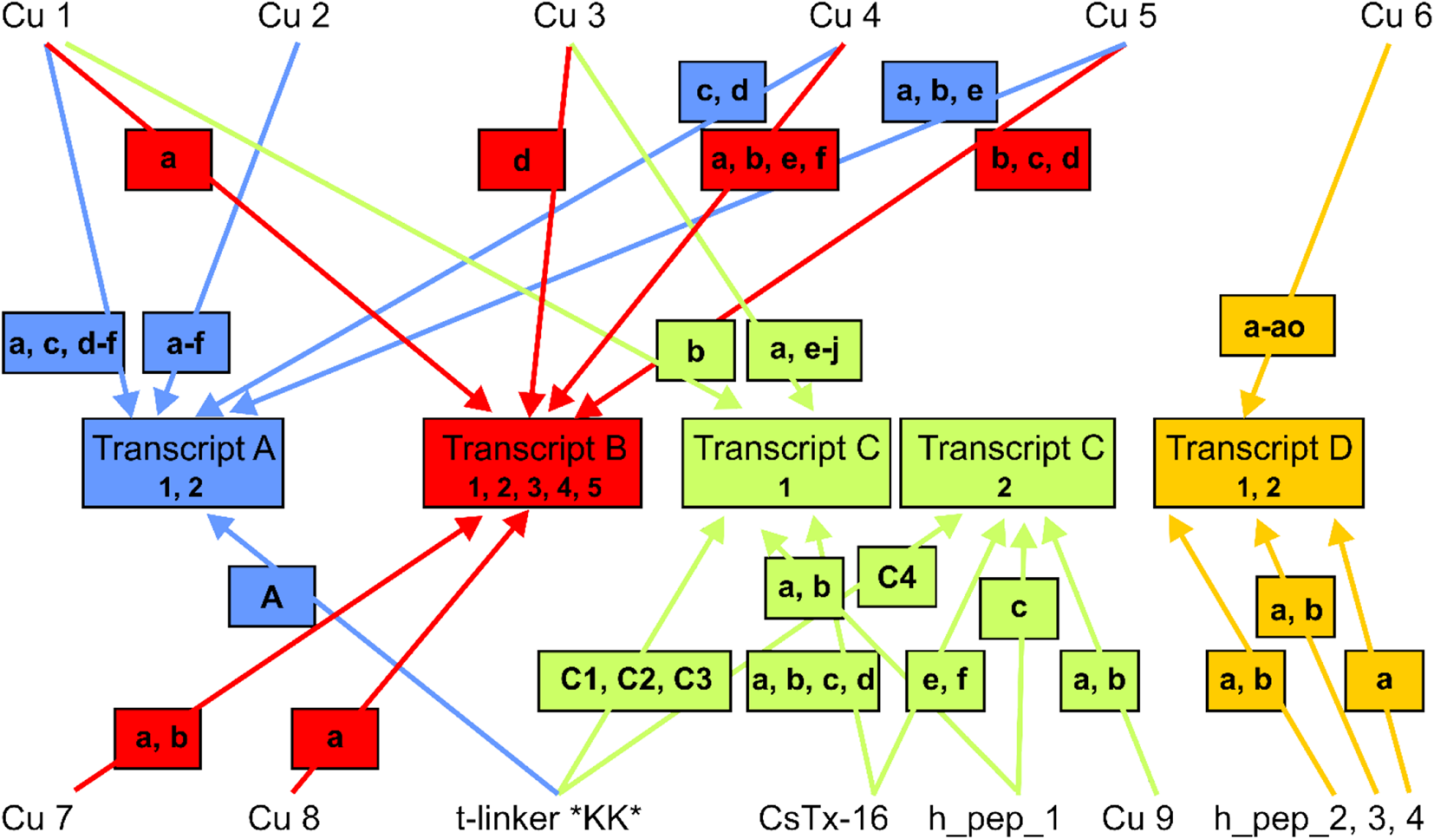
Overview on the composition of the transcript families (**A**–**D**) with different cupiennin families. Transcript families are encoded in different colors. Colored arrows coming from the peptide families indicate their occurrence in different transcript families.

Four transcript families encode all LPs. Transcript A and B families appear similar in their composition, even though different members of the Cu families are encodes. Especially the succession and the presence of different or identical peptides (e.g., Cu 2a, transcript A), separated by different or equal linkers, is variable. Additionally, several short cuts between different peptides have been identified in different transcripts (in detail: S1A-E Fig). In transcript A, members of four different Cu families were identified: Cu 1a, c, d-f, Cu 2a-f, Cu 4c, d, and Cu 5a, b, e. Likewise, in transcript B, six different Cu families were identified: Cu 1a, Cu 3d, Cu 4a, b, e, f, Cu 5b, c, d, Cu 7 a, b, and Cu 8a. Interestingly, only Cu 1a and Cu 5b occur in both transcript families, but apart from this, different variants of the same Cu family exist (Fig. 3).

On the first view, the C-terminal transcript structures of the A and B families are not so clear, but overlapping linker/peptide/linker/stop codon analysis provides evidence that transcript A1 ends after Cu1a C-terminal with a t-linker. The t-linker starts with an iPQM1 but misses a PQM and after 12 amino acid residues, comparable to the length of all linkers in the transcript A1 and B families, a stop signal appears, followed by two Lys residues and again a stop signal. However, the transcript B families exhibit C-terminally after Cu 1a a specific linker before the terminal peptide Cu 7a, b, followed by a stop signal. This linker is characterized by an iPQM2–PQM structure and is also composed of 12 amino acid residues. Supportive for this data interpretation is the fact, that Cu 1a and Cu 1f peptides of transcript A1 exhibit a silent mutation for Tyr^28^ in third position (TAC). This mutation is additionally present in Cu 1a and Cu 1f which are followed by a t-linker. In contrast to this, Cu 1a identified C-terminally in family B, exhibits TAT as cDNA code for Tyr^28^ (S1A and S1B Fig, S2A Fig).

Comparably, Cu 5b peptides are identical in both transcript families only on protein level. Within transcript A, four different variants have been identified and a fifth variant occurs in the transcript B family. Seven point mutations in third position (transition as well as transversion) are the driving force for that variation (S1A and S1B Fig, S2B Fig).

Transcript B and C1 families share on protein level an identical signal peptide. However, on nucleotide level only signal peptides of transcript B subfamilies are identical and differ by the signal peptide of C1 in three point mutations in third position. Cu 1 (transcript A, B) and Cu 3 (transcript B) families are present in transcript C1, but the expressed peptide variants, Cu 1b and Cu 3a, e-j, are unique in transcript C1.

Transcript C1 and C2 families are in so far unique, as LPs occur that exhibit as specific feature one Cys residue in the middle of the sequences (Fig. 2). Taking previous proteomic results into account^15^, always two succeeding peptides are connected by a specific linker (iPQM2–PQM structure, 11 amino acid residues), and posttranslationally processed to a family of two chain peptides, CsTx-16, by the formation of one disulfide bridge. In the transcript C1 family, after Cu 3a the CsTx-16 chains a1, 2, 3 are followed by the CsTx-16 chains b1, 2, and 3. Likewise, the CsTx-16 chains c1, 2 are followed by the CsTx-16 chains d1, 2. A short variant is given in transcript C2, where after a short propeptide the CsTx-16 chains e and f appear, followed by a so far not identified peptide family, Cu 9. The C-terminal structure of the transcript families C1and C2 is comparable to the structure identified in transcript A. After the chains CsTx-16 d1, 2 and Cu 9a, b, t-linkers (iPQM2–PQM, 16 amino acid residues) terminate the transcripts with a stop signal, followed by one or two Lys residues and again a stop signal (Table 1, S1C and S1D Fig).

Taking the C-terminal structure of transcript B into account, the transcript may hypothetically also be terminated by a further peptide group and a stop codon. Such a structure was identified in a very low read amount for transcripts C1 and C2, which is caused by an indel mutation (addition of one adenine) in the t-linker after RSFDSN, resulting in a frame shift. As consequence, the t-linker ends after 21 amino acid residues with a PQM, followed by the hypothetical peptides 1a, b, c, and a stop codon (S1C and S1D Fig, S3 Fig).

The transcript D family shows no relationship to all above described transcript families in terms of comparable peptides or linkers. Here, six only slightly different and transcript family-specific linkers (iPQM2–PQM, 13 amino acid residues) connect 17 different peptides, all belonging to the Cu 6 family (Table 1). Cu 6a, c, h, and i, are identified one to three times at different positions within the transcript. C-termination is done with Cu 6f, followed by a twofold stop signal. Comparable to the C-terminal situation of transcript C, an indel mutation (here a subtraction of one adenine) in Cu 6f, when compared to h_pep_2a, occurred. Through the corresponding frame shift, this created three further hypothetical peptides (h_pep_2ab, h_pept_3ab, and h_pep_4ab) with high read numbers. Interestingly, no further linker could be identified between them, but two dibasic ArgC protease cutting motifs “RK” and a PQM motif, were identified (S1E Fig, S4 Fig).

### Cupiennin families

Nine different peptide families were identified by 454-seq and NGS. With the exception of the Cu 5 and 9 families, all other families exhibit a C-terminal Gly residue, which is used for C-terminal amidation of the mature peptides as deduced from proteomic data. For most peptides, α-helical parts could be theoretically calculated. Furthermore, four hypothetical peptide families were detected through transcripts C and D analysis. With NGS, 134 LPs were identified and 39 peptides of them are identical to the 84 peptides identified by 454-seq. In total, 179 small cationic LPs were identified in both transcriptomes (Table 2, S1A Table).

**Table 2:**
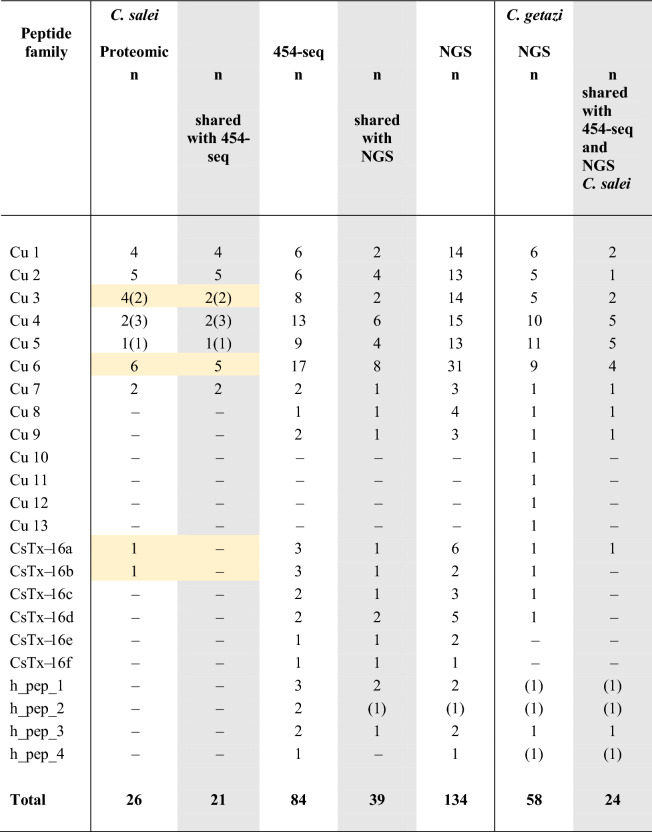
Overview on identified LPs of *C. salei* by 454-sequencing and NGS as well as of *C. getazi* by NGS sequencing. Additional numbers in brackets refer to truncated variants identified by proteomics, not added to the total number. Here single chains of CsTx-16 are given. Highlighted in orange are LPs identified only in proteomics and highlighted in grey are shared LPs.

The Cu 1 family is the best investigated cytolytic peptide family of *C. salei*. All known peptides (Cu 1a, b, c, d)^15^ were identified in the transcriptome by 454-seq, as well as further members of the Cu 1 family, Cu 1e and Cu 1f by 454-seq, and Cu 1g to Cu 1r by NGS. The peptides are composed of 36 amino acid residues, including the Gly for C-terminal amidation of the mature peptides. They have molecular masses between 3717 and 3931 Da, and are highly cationic with pIs between 9.78 and 10.54. The more hydrophobic N-terminus is connected by a sixfold repeat of four amino acids, starting always with Lys, and ends in a more hydrophilic C-terminus. All identified Cu 1 variants show predicted α-helicities between 44 and 75%.

**Figure 4:**
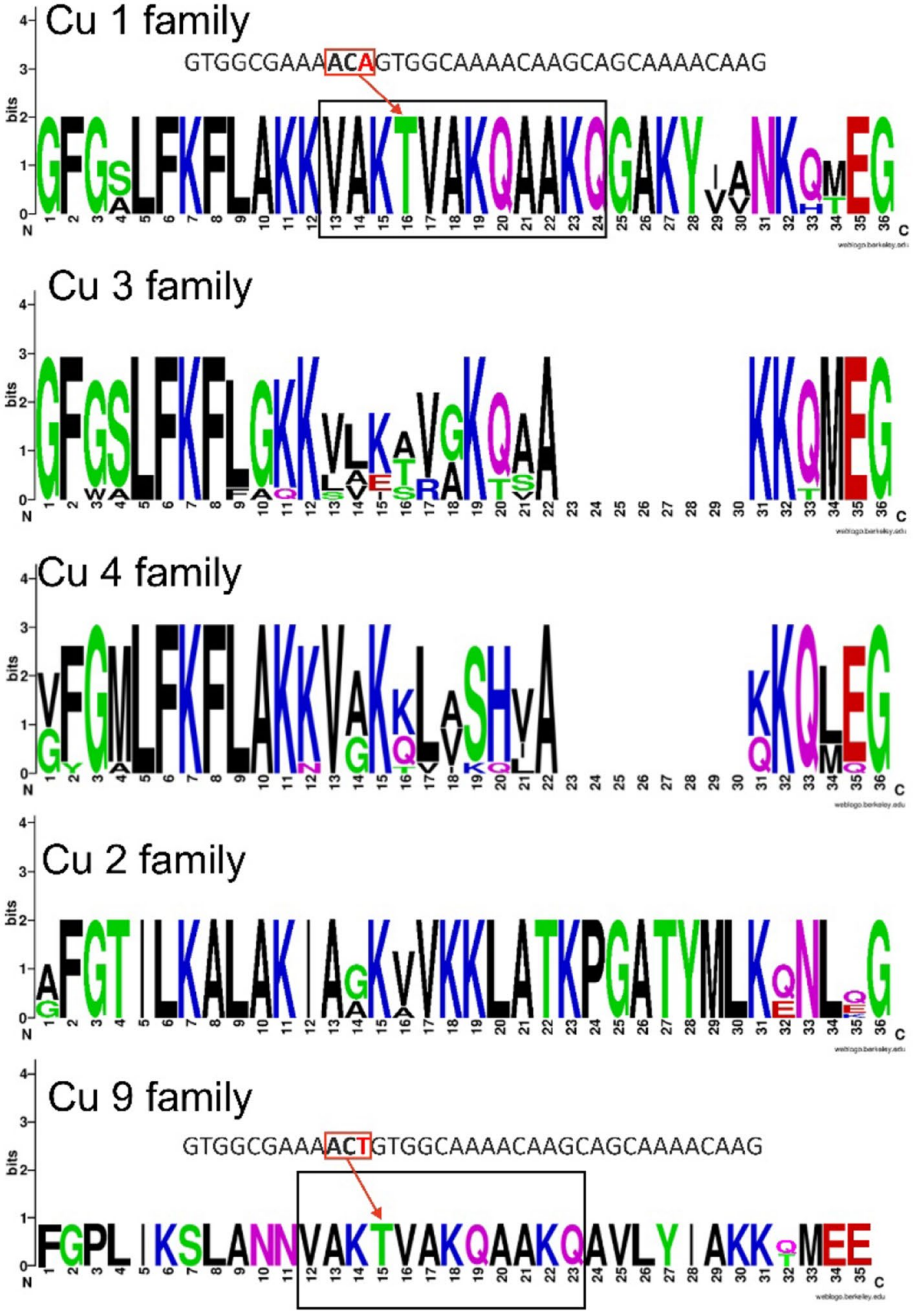
Amino acid sequence logos of related cupiennin families. The relative frequency of each amino acid residue at a certain position of different cupiennin families is given as sequence logo. Cationic amino acids are colored in blue, anionic amino acids in red, the corresponding C-terminal amid variants in pink, polar amino acids in green, and hydrophobic amino acids in black.

The Cu 3 family (28 amino acid residues, 2969–3109 Da) differs only in the C-terminal part from the Cu 1 family, where the last two C-terminal repeats are missing. Comparable to the Cu 3 family, the Cu 4 family (28 amino acid residues, 2940–3184 Da) misses likewise the last two repeats of Cu 1, and confirms the truncated variants obtained by proteomics studies. All peptides are characterized by pIs mainly above 10, a high net charge between + 4 and + 8, and predicted α-helical structures between 46.4 and 78.6% (S1A Table). An overview on the sequence logo^26^ of the Cu 1, Cu 3, and Cu 4 families highlights the related peptide structures (Fig. 4).

The Cu 2 family (36 amino acid residues, 3719–3887 Da) mainly differs in the C-terminal part from Cu 1, apart from that the physical and chemical parameters are comparable to those of the Cu 1, 3, and 4 families. Even though the Cu 9 family (35 amino acid residues, 3775–3802 Da) differs in its N- and C-terminal part from Cu 1, a sequence of 12 amino acid residues is on nucleotide level nearly 100% identical, except a point mutation in third position of Thr (Fig. 4).

**Figure 5:**
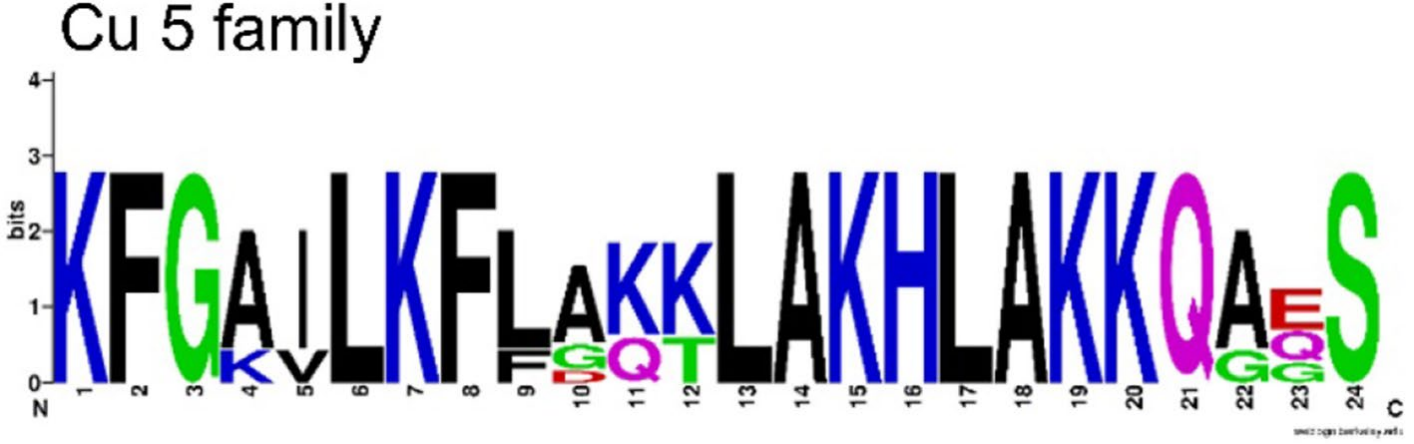
Amino acid sequence logo of the Cu 5 family. The relative frequency of each amino acid residue at a certain position of different cupiennin families is given as sequence logo. Cationic amino acids are colored in blue, anionic amino acids in red, the corresponding C-terminal amid variants in pink, polar amino acids in green, and hydrophobic amino acids in black.

The Cu 5 family is mainly present in transcript C, but also contains Cu 3d that primarily belongs to transcript B. In opposite to most other identified peptide families, the Cu 5 family exhibits no C-terminal Gly, implying no C-terminal amidation of the mature peptides. With 24 amino acid residues, molecular masses between 2585 and 2713 Da, and pIs nearly all above 10, these peptides are characterized by the alternating sequence of cationic amino acid residues and non-polar and hydrophobic amino acid residues (Fig. 5).

The Cu 6 family (24.3%) is the most diversified (20–22 amino acid residues) LP family. It comprises 17 different peptides identified by 454-seq (Table 2, S1A Table), five of them have been elucidated on proteomic site^15^, and further 31 peptides by NGS, whereas eight of them are identical to peptides identified by 454-seq. In addition, two related peptides (h_pep_2ab, 454-seq) variants were identified, in total 42 peptides. These peptides exhibit molecular masses between 2458 and 2826 Da, pIs between 9.52 and 11.70, and 13.6–35.0% of their amino acid residues are positively charged. Strikingly, the first third of the sequences are characterized by hydrophobic amino acid residues which are mainly connected by a short α-helical part to the polar/charged C-terminus. Except for Cu 6f and h_pep_2ab, all other peptides are C-terminally amidated.

**Figure 6:**
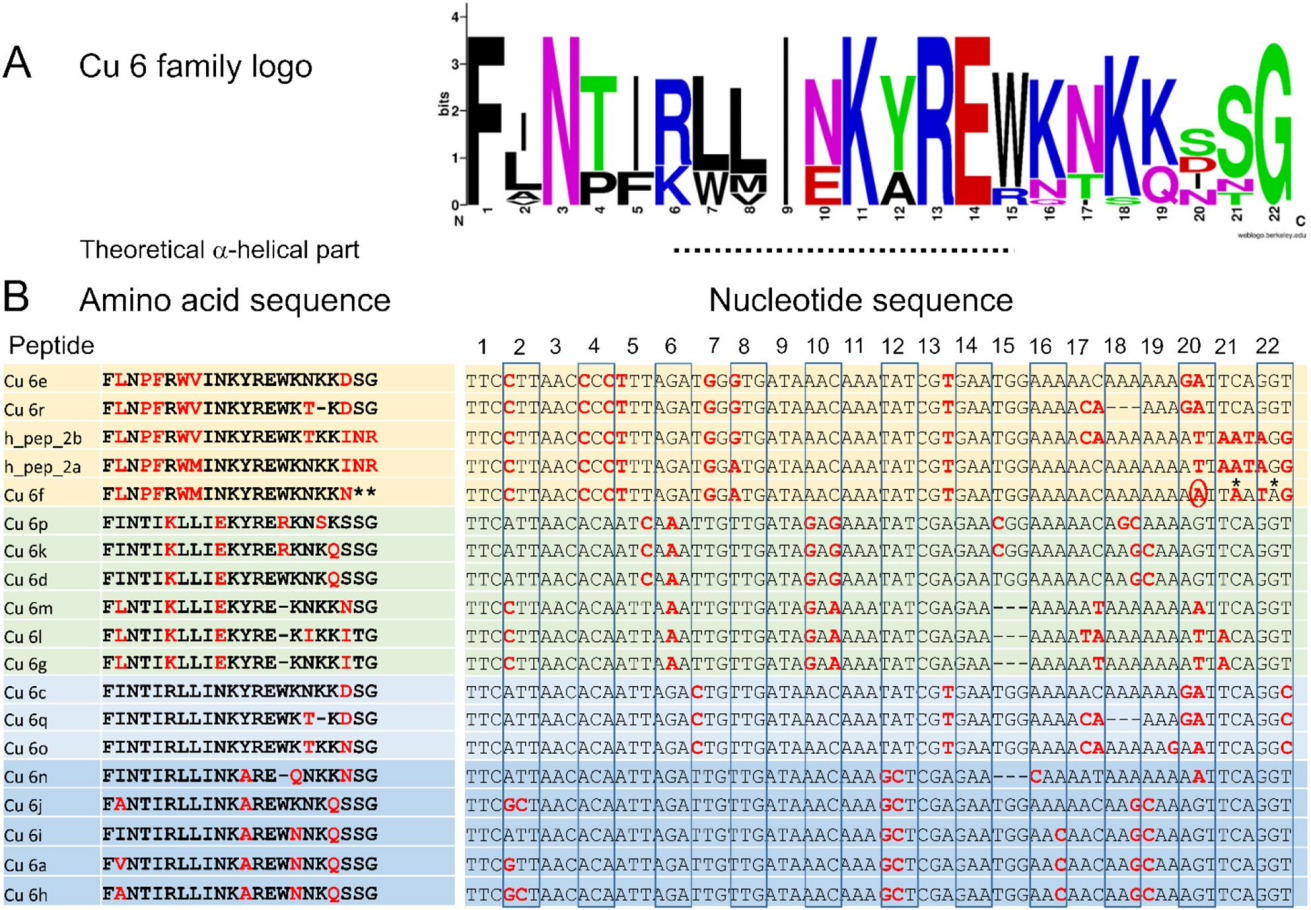
Amino acid sequence logo and the nucleotide sequences of the Cu 6 family variants identified by 454-seq. (**A**) The relative frequency of each amino acid residue at a certain position of different cupiennin families is given as sequence logo. Cationic amino acids are colored in blue, anionic amino acids in red, the corresponding C-terminal amid variants in pink, polar amino acids in green, and hydrophobic amino acids in black. The theoretical α-helical part of the peptides is indicated as a dashed line. (**B**) Amino acid and nucleotide sequence of different Cu 6 sub-families are colored in orange, green, light blue, and blue. Mutations are highlighted in red, stop signals are marked with asterisks, and deletion of an adenine in Cu 6f, resulting in h_pep_2ab, is designated with a red circle.

An overview on the sequence conservation within the Cu 6 family and the relative frequency of each amino acid residue at a certain position is given as sequence logo^26^ (Fig. 6A). Furthermore, the mutations behind this variety of peptides are elucidated on cDNA level as deletions of three base pairs (Cu 6 m, l, g, q, n, r), and different substitutions in nearly half of all nucleotide positions (Fig. 6B), equally distributed over the entire sequences.

The C-terminally identified Cu 7 family in transcript B is confirmed by earlier proteomic data^15^. With 18 amino acid residues these peptides are the shortest cationic LPs so far identified in spider venoms. About one third of all amino acid residues are referring to Arg or Lys and in the middle of the peptides short α-helical structures, mainly of cationic amino acid residues, are predicted (S1A Table).

**Figure 7:**
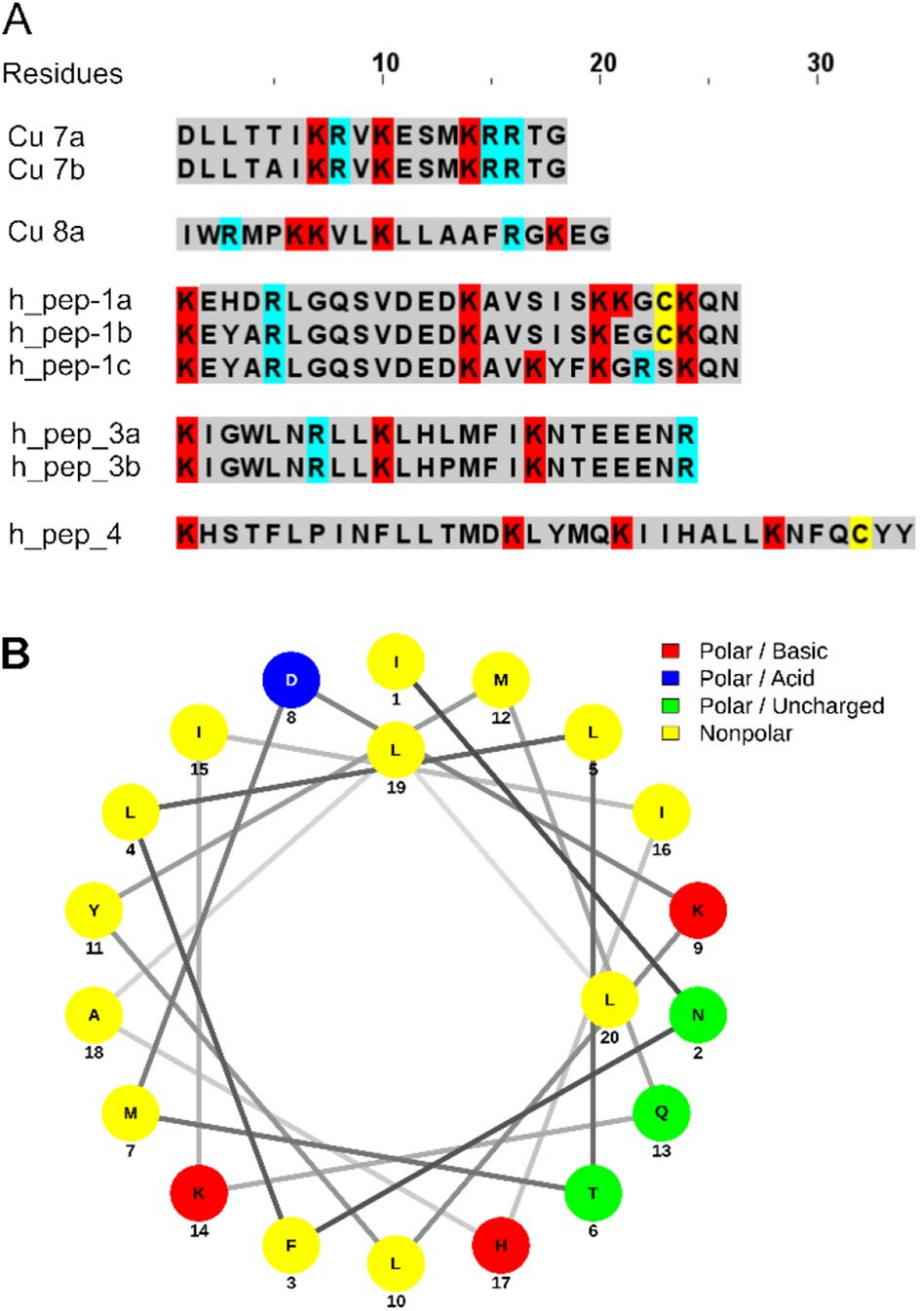
Overview of Cu 7, 8, and hypothetical peptides families identified by 454-seq in the transcriptome of *C. salei.* (**A**) Amino acid sequences of Cu 7, 8, and of the hypothetical peptide families 1, 3, and 4. Cationic amino acids are marked in red (Lys) and blue (Arg), and Cys in yellow. (**B**) α-helical wheel projection^27^ of the proposed α-helical part (Ile^8^–Leu^27^) of h_pep_4.

A further short LP family is Cu 8 (transcript B), which is composed of 20 amino acid residues and about one third of all amino acid residues are Lys or Arg. In the middle of the peptide, a short α-helical structure is predicted. Interestingly, only four peptide variants were identified (Cu 8a, 454-seq; Cu8b, c, d, NGS), albeit both peptides are the first peptides after the propeptide (Fig. 7A, S1A Table).

Only on transcriptomic site identified, the Cu 9 family consists of two variants (Fig. 4). Strikingly, within the α-helical part of the peptides, a sequence of 12 amino acid residues (Val^12^ to Gln^23^) is identical with a sequence part of Cu 1 (Val^13^ to Gln^24^), except one silent mutation in Thr^15^.

### Hypothetical peptide families and t-linkers

Usually, a transcript ends C-terminally with a stop codon after the encoded peptide, as shown for all neurotoxin transcripts, identified in the venom gland transcriptome of *C. salei*^20^. After the analysis of the C-terminus of transcript A and B families, two possibilities were found. The first possibility results in a C-terminal peptide, which is connected by an unusual linker (RSLNFMDNEEQR) with the previous peptide, and followed by a single stop codon e.g., Cu 7 (transcript B). The second possibility exhibits a t-linker after the last peptide, lacking a C-terminal PQM motif, followed by a stop signal, two Lys residues and again a stop signal (transcript A). When we apply these two variants to all other identified transcript families, more hypothetical peptides can be identified for the transcript C families. Transcript C1 and C2 families exhibit after an unusual long linker of 21 amino acid residues (Table 1), a comparable peptide, named hypothetical peptide h_pep_1a, b, c, followed by a stop signal (Fig. 7A). These peptides (2900 Da, 26 amino acid residues) have been identified only in a low read number (2.7% of both C-terminal endings) and probably play no important role (S1C and S1D Fig).

Transcript D exhibits also two C-terminal processing possibilities. In contrast to transcript A and C, the first possibility is characterized by Cu 6f, which is followed by two stop signals, and no t-linker. The second possibility comprises hypothetical peptide variants, which have been identified in a read number comparable to the C-terminal peptide Cu 6f. Theoretically, through the presence of two dibasic RK motifs, the peptide is further posttranslationally processed to three separated peptides, h_pep_2a, b (Fig. 6B), h_pep_3a, b (2969–3040 Da, 22 and 24 amino acid residues), and h_pep_4 (4177 Da, 34 amino acid residues) (Fig. 7A). Such hypothetical peptides were also identified by NGS. All three peptide families may adopt in their middle part α-helices, as predicted theoretically. The α-helical wheel projection of the proposed α-helical part of h_pep_4 exhibits a well separated hydrophobic site from a more positively charged area (Fig. 7B, S1E Fig).

### Two-chain peptides: CsTx-16

**Figure 8:**
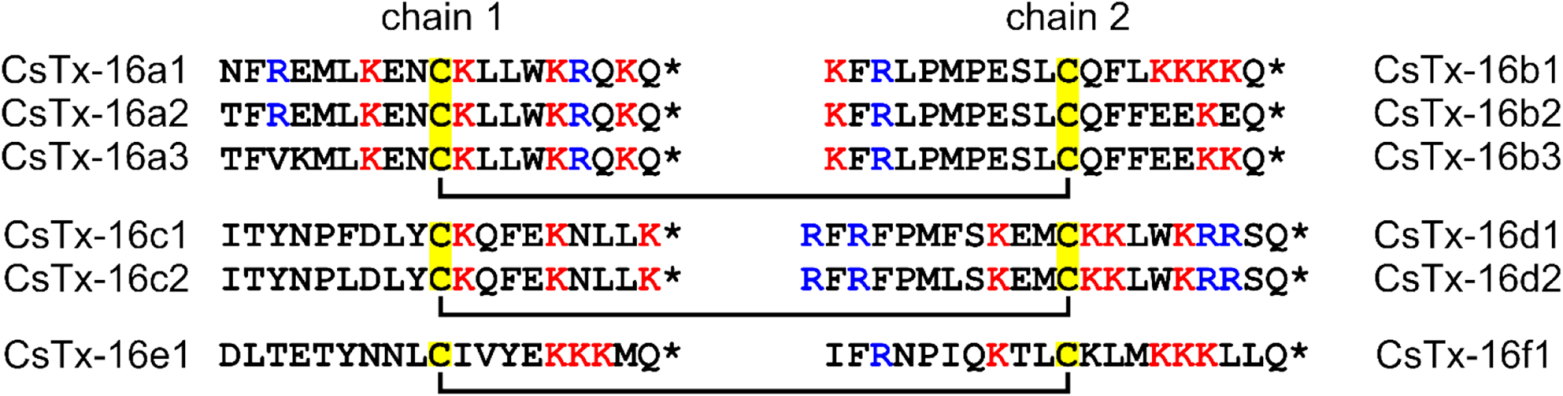
Overview on different CsTx-16 two-chain peptides. Three main variants of CsTx-16ab, CsTx-16cd, CsTx-16ef, and some sub-variants are presented. Cationic amino acid residues are colored in blue (Arg) and red (Lys) and Cys is highlighted in yellow. The C-terminal amidation is indicated with asterisks (*).

With 13.5%, the CsTx-16 variants represent, after Cu 6, the second most frequent linear peptide family identified in the transcript families C1 and C2. CsTx-16 two-chain peptides are the result of a posttranslational modification in terms of a disulfide bridge formation between two specific linear peptides, which are connected by a specific linker (Fig. 8). Even though we could not identify exactly the previously published sequence data [UniProtKB–B3EWS9] based on Edman degradation^15^, this data allows the mapping of the corresponding linear peptides. Interestingly, a synthesized variant of this CsTx-16 variant is able to adopt an α-helical conformation in the presence of trifluoroethanol^28^. All peptides are C-terminally amidated and exhibit a high content of basic amino acids (15–37%). Chain 1 is composed of 20 amino acids and chain 2 varies between 20 and 22 residues. The main two-chain peptide variant is CsTx-16a1b1 (32.6%/28.9%), followed by CsTx-16c1d1 (19.0%/14.6%).

### Insecticidal activities of selected cupiennins and CsTx-16

The insecticidal activity of different cupiennins was investigated in a bioassay with *Drosophila melanogaster* and the lethal dose, where 50% of the insects die of intoxication after 24 h of injection, calculated. Variants of the cupiennin 1 family showed the lowest LD_50_ concentrations between 4.7 and 6.4 pmol/mg fly, thus these substances are most toxic. The shorter LPs such as Cu 3a, d, and Cu 4a, were with 12.4–38.5 pmol/mg fly less toxic. The toxicity decreased from Cu 6a, b, and d to LD_50_ concentrations of 23.2–78.0 pmol/mg fly and the lowest toxicity was determined for Cu 8 and Cu 7b (Table 3). Interestingly, the two-chain peptides CsTx-16a1b1 and CsTx-16c1d1 are about ten-fold less toxic than variants of the Cu 1 family. When comparing the insecticidal activity of the cupiennins with neurotoxins isolated from *C. salei*, only CsTx-1 is by a factor of 10 more active than Cu 1b. However, the determined insecticidal activity (flesh flies) of cyto-insectotoxin 1a (CIT 1a), one of the longest linear peptides so far described for spider venoms (*Lachesana tarabaevi*)^18^, is in the same range as CsTx-1.

**Table 3:** Insecticidal activities of selected cupiennins, CsTx-16 variants, neurotoxins CsTx-1, CsTx-9, and CsTx-13, and cyto-insectotoxin CIT1a. Different concentrations of peptides were dissolved in 0.1 M ammonium acetate, pH 6.1, and 0.05 µl was injected into *Drosophila* flies. The lethal dose (LD_50,_ pmol/mg fly), where 50% of the flies die after 24 h, was calculated with PRISM Vers. 6.07. * Data are from^14^, ** from^25^, *** from^18^, using flesh flies.

Peptide	LD_50_	95% Confidence Intervals
	(pmol/mg fly)	
Cu 1a*	5.9	4.2–8.3
Cu 1b*	4.7	3.9–5.7
Cu 1d*	6.4	5.4–7.6
Cu 3a	18.7	17.11–20.4
Cu 3e	38.5	34.2–43.3
Cu 3d	12.7	11.3–14.1
Cu 4a	12.4	11.0–14.1
Cu 6a	78.0	70.3–86.6
Cu 6b	39.5	35.6–43.9
Cu 6d	23.2	21.4–25.2
Cu 7b	438.1	423.6–453.0
Cu 8	159.7	154.7–164.8
CsTx-16a1b1	63.5	58.1–69.3
CsTx-16c1d1	53.6	49.1–58.5
CsTx-1**	0.53	0.515–0.555
CsTx-9**	45.54	43.30–47.78
CsTx-13**	111.2	105.5–116.9
CIT1a***	~ 0.6	

### Phylogenetic aspects

Is the high diversity of LPs identified in the venom gland transcriptome of *C. salei,* species or genus specific? We analyzed the venom gland transcriptome of *Cupiennius getazi*, the sister species of *C. salei*. As expected, from every cupiennin family at least one identical LP on peptide level was identified in both species. Beside 24 shared LPs, additional 30 new variants of known cupiennins, and four new peptides, Cu 10, 11, 12, and 13 were found (Table 2, S1B Table). Furthermore, signal peptides starting the transcript families A, B, C, and D of *C. salei* are identical (transcript B and C) or very similar (transcript A and D) with the corresponding transcript families in *C. getazi*. Both variants of the striking C-terminal t-linkers identified in the *C. salei* transcriptome are present in *C. getazi*. Therefore, we assume that this unique importance of LPs in the venom and the corresponding processing mode is typical for the genus *Cupiennius*.

Though we could not find the here described peptides in the closer related families of lycosids, pisaurids, and oxyopids, the highest similarity could be detected in lycosids (unpublished results L. Kuhn-Nentwig).

## Discussion

Over 315 million years, spider venom evolution was driven by two crucial factors, constant selective pressure on the efficiency of its components as well as optimization of their biosynthesis costs. The oldest strategy identified in mygalomorph spiders refers to the synthesis of hundreds of single, target specific neurotoxins, with targets usually being ion channels. Recruitment of a single ancestral DDH and/or ICK gene into the venom gland of the Australian funnel web spiders, followed by multiple gene duplications, diversification, and selection of neurotoxins by adaptive evolution, but also intragene duplications, are the key for the production of such neurotoxins^29^. This implies high metabolic costs for the spider, as one peptide precursor results in only one specific mature neurotoxin, for one specific target. The more such neurotoxins are available in venom, the higher the chance, to subdue a large diversity of prey types and to minimize the probability of resistance evolving against a specific neurotoxin, but also the higher the production costs.

An alternative strategy becomes apparent in some modern araneomorph spider families, through the additional production of less specific small, cationic and insecticidal LPs, paralleled by a concurrent reduction of the number of ICK-containing neurotoxins^10,20^. Besides *C. salei*, only for two further spider families, zodariids and oxyopids, very limited knowledge about the corresponding transcript structures is available. An analysis of the oxyopid transcript structure of oxyopinin 1a,b, and c (tr|A0A5J6SIH8, tr|A0A4D6Q2Y9, tr|A0A4D6Q7V4) shows simple precursors, and the transcript structures of oxyopinin 2 (tr|A0A5J6SEB1_OXYTA; submitted by Vassilevski, Kozlov, Grishin, 2018) highlights a binary precursor structure resulting in oxyopinin 2a and 2b^13^. As reported for the zodariid *L. tarabaevi*, latarcins are mainly synthesized as simple precursors and a minority as binary precursors, as one precursor results in one or two active peptides, or in the case of a complex precursor, in five to six LPs, all separated by linkers^10^. This situation is comparable to those in the long transcript families A-D in *C. salei*. The optimization strategy as realized in *C. salei* implies longer and more complex transcripts, nearly no simple or binary precursors, and the consequent separation or linking of LPs by specific linkers. Mainly four different transcript families are responsible for the expression of nine LP families and four hypothetical LP families, resulting in 179 different mature LPs as identified by 454-seq and NGS. This is for *C. salei* nearly a three-fold higher output of active peptides when compared with its identified 81 transcripts of neurotoxins resulting in a total number of 54 neurotoxins^20^. Such a strategy reduces metabolic costs considerably and makes *C. salei*, according to current knowledge, the most successful modern spider in terms of diversification of venomous components, especially of LPs, in its venom.

Previous investigations of Cu 1a and Cu 1d highlight these LPs as “cytolytic all-rounders”, because they are active on bacteria, eukaryotic pathogens such as trypanosomes and plasmodia, the causative organism of malaria. Additionally, diverse human blood cells, human leukemic cells and human tumor cells are destroyed by these peptides^14,30^. With such properties, cupiennins are comparable to versatile, membrane active LPs identified in lycosids^31,32^ and zodariids^9^.The insecticidal activities of cupiennins differ by a factor of 100 when comparing Cu1b and Cu 7b. This indicates that the targets might be different. Hence, *C. salei* always endeavors to optimize the venom activity by synergistic activities^20,22,24,33^ in which the toxicity on insects is potentiated, e.g. by neurotoxin merging^25^.

As mentioned above, gene duplication, diversification and intragene duplication are only a part of the explanation of such an extraordinary amount of identified LPs. Spider DNA is characterized, in contrast to other arthropods, by short exons and long introns^34^. It is tempting to speculate that alternative splicing of such genes, gene-based combinatorial peptide library strategies and the induction of a hypervariability-generating mechanism^35^, as visible for example in the Cu 6 family (Fig. 6), are the driving force behind this tremendous diversity of LPs (Fig. 1).

There is no obvious relationship between the length of the mainly anionic propeptides and the following number of cationic LPs in *C. salei*. However, up to three different iPQMs and four PQM motifs were identified therein, which allows a fast degradation of such peptides. Comparably, some of the propeptides of latarcins also exhibit iPQM and/or PQM structures^9^. In contrast, the above mentioned transcripts of oxyopids exhibit the longest propeptides (52 and 54 amino acid residues), no iPQM and only one PQM structure, but also the longest LPs (oxyopinin 1a, b, c) composed of 48 amino acid residues^13^.

Linkers between different LP precursors are multifunctional. During the translation of long transcripts, they connect and simultaneously separate highly cationic peptides, based on their high content of negatively charged Glu and Asp residues. Their specific N-terminal iPQM and C-terminal PQM structures (Table 1) provide access to possibly specific proteases, which (1) release different LPs and (2) facilitate the specific formation of disulfide bonds between well-defined pairs of linear peptides, each containing one Cys, resulting in the CsTx-16 family as describe here for the first time. Such a production mode allows a higher number of different combinations of the corresponding variants of the single chains of CsTx-16ab, cd, and ef. We can assume that the iPQM structure and the length of linkers may influence the kinetics of the proteolytic activity, because the linkers before and after the corresponding peptide pairs have to be cut before the formation of the disulfide bond and the linker between the corresponding single chains after the disulfide formation.

The here proposed maturation of CsTx-16 peptides, highlights a second biosynthesis pathway for two-chain peptides. So far, two-chain neurotoxins as CsTx-8, 12, and 13 from *C. salei*^20,23^, or omega-aga-1A from *Agelenopsis aperta*^36^ are the result of a specific proteolytic activity of a PQM-protease on a single peptide^17^. After the formation of disulfide bonds within these peptides, an iPQM and PQM structure is identified by a specific protease, resulting in the described two-chain peptides.

Length and structure of linkers seem to be at least specific for a spider genus, if not for a family. From both *Cupiennius* species investigated here, linkers are identical, they are mainly composed of 13 amino acid residues, and in the context of CsTx-16, they consist of 11 amino acid residues. *L. tarabaevi* exhibits linkers composed of eight, nine or ten amino acid residues^10^ and in *Oxyopes takobius* a linker of 25 amino acid residues connects oxyopinin 2a and 2b.

Strikingly, a high number of reads, especially in transcript A, exhibits C-terminally a t-linker, followed by a peculiar arrangement of two stop signals, separated by two Lys. The function of such t-linkers is still enigmatic. They show sequence similarities to a part of the EF-hand 2 low-affinity calcium binding site of the neuronal calcium sensor protein recoverin of vertebrates^37^, but also to a part of the DEAD/DEAH box helicase of bacteria (WP_146269254.1:758–769, *Mucilaginibacter* sp.). Possibly, this indicates venom compounds with yet unknown function.

In conclusion, we see a considerable application potential of the expression strategy of *C. salei* concerning such long inactive peptide chains. They can be used as blueprint for the concurrent recombinant expression of selected LPs in bacteria, which can be further processed in a second step with the help of the PQM protease^17^. Many LPs are active on a large number of negatively charged membrane systems and also not identified targets^30^, present in all living organisms. These properties make them of interest for the pharmaceutical industry^5,9,38^. Also intracellular effects through a possible uptake of such peptides into different cells in terms of efficient intracellular drug delivery^39,40^ should be investigated.

## Material and methods

### Spider maintenance and cDNA libraries of venom glands

*C. salei* (own spider stock since 1990) and *C. getazi* (originated from Costa Rica; A. Leetz) were laboratory bred and adult spider were used for the generation of transcriptomes. A first cDNA library of *C. salei* venom glands was generated by 454-sequencing (Skuldtech, Montpellier, France)^19^. The venom glands of 20 adult female spiders were dissected after electrical milking at different time intervals (24, 48, and 62 h and 8 and 14 days) and stored in RNAlater (Qiagen). The samples were sent to Skuldtech and from pooled venom gland material RNA was extracted. The quality and quantity of RNA were tested by Nanodrop and Bioanalyser 2100 Agilent and 15 µg were used for EST library construction. Sequencing runs yielded 460,000 ESTs and sequence assembly was performed by de novo using MIRA_2 (V2.9.25 with enhanced 454 support) using 98% of homology. The assembly resulted in a total of 34,107 contigs and 202,877 single sequences.

Later, further cDNA libraries of *C. salei* and *C. getazi* venom glands were generated on an Illumina HiSeq3000 platform (University of Bern, Switzerland). Briefly, from 16 adult female *C. salei* spiders and 31 adult *C. getazi* (18 male and 13 female) species the venom glands were dissected after milking at different time intervals (1 h, 4 h, 8 h, 12 h, 24 h, 48 h, 62 h and 7 days for *C. salei* and 4 h, 8 h, 12 h, 24 h, 48 h and 72 h for *C. getazi*) and stored in RNAlater. The extraction of total RNA was done by an in-house protocol combining phenol/chloroform extraction with the RNeasy mini kit (Qiagen). The quality and quantity of RNA was assessed by Nanodrop, the Qubit RNA BR assay kit (Qubit 2.0 fluorometer; Thermo Fisher Scientific) and by an advanced analytical fragment analyzer system (fragment analyzer RNA kit, DNF-471, Agilent). One µg of RNA was used for each cDNA library preparation with the Illumina TruSeq-stranded mRNA prep kit. For further sequencing double barcoding and selected fragments with lengths between 300 and 600 bp (Pippin HT system, Sage Science) were used. To avoid cross contaminations between cDNA libraries of different spider venom gland transcriptomes, both libraries were multiplexed (25% per lane) timely independent, and with other non-arthropods, mostly genomic libraries of vertebrates. Assemblage of the resulting reads was done using Trinity version 2.1.1 (*C. getazi*) and version 2.5.2^41^ (*C. salei*), both with default settings^17,21^. The assembly of *C. salei* clusters (22.6 millions) resulted in 92,023 contigs and of *C. getazi* clusters (41.8 millions) in 126,472 contigs.

### Transcriptome analysis of 454-seq data and assemblage of transcript A, B, C1, C2 and D families

All so far on proteomic site identified cupiennins and CsTx-16^15^ were used to search with BlastP (E-threshold, 0.0001) against the above mentioned 454-transcriptome (454-seq). Firstly, the obtained contigs were analyzed and sorted in terms of identical peptides, belonging to different cupiennin families. Secondly, a further sorting was done by taking specific linkers, which are in N- or C-terminal position to the peptides, into account. A linker sequence is characterized N-terminally by a PQM and C-terminally by an iPQM motif. With the obtained specific N-terminal- or C-terminal linker information of the peptides, peptide chains were assembled and elongated in both directions until N-terminally the signal peptide or C-terminally a stop codon was identified.

For the assemblage of the transcript families A, B, C1, C2, and D (see S1A-E Fig), only transcriptomic data from 454-seq with the following conditions were used: every peptide/linker unit had to be identified at least in two contigs, composed at least of five reads. Mainly completely identified peptide sequences, built at least of a linker/peptide/linker or a peptide/linker/peptide structure, were used for overlapping elongation of the protein sequence. Counting of the identified peptides at a certain position in the transcript families was done by read counts of the corresponding contigs. For the determination of the content of individual peptides, reads of only fully premature sequences including a C-terminal glycine for amidation, which is characteristic for several cupiennin families (S1 Table), were counted (Fig. 1, S1A-E Fig). The transcriptomic data analysis workflow, and especially the manual construction of the transcript families, is given in detail in (S5 Fig).

#### Identification of LPs in Illumina HiSeq3000 transcriptomes

All known LP sequences obtained earlier by Edman-sequencing of purified peptides from *C. salei* venom and of 454-seq identified contigs, encoding signal peptides, propeptides and LPs including the linker regions, were used as query and blasted against the Illumina 3000-seq venom gland transcriptomes of *C. salei* and *C. getazi*. Analysis of the obtained contigs were focused on the identification of identical, related and new LPs.

LPs, which were accepted as identical or related peptides had to be identified in one contig exhibiting full N-terminal and C-terminal peptides linkers, or at least in two contigs, exhibiting at least 12 bps of both linkers (iPQM and PQM motif). Complete new peptides were characterized by full N-terminal and C-terminal linkers and at least of two contigs. Additionally, all transcriptomes were blasted against a set of so far published small LPs identified in zodariids, oxyopids and lycosids (S5 Fig).

#### Phylogenetic aspects

All identified LPs from the Illumina3000 transcriptome of *C. getazi* were sorted to the corresponding cupiennin families created for *C. salei*. Alignments of peptide, signal and propeptide sequences belonging to different cupiennin families of *C. salei* and *C. getazi* were done by Clustal omega (www.ebi.ac.uk)^42^. New peptide families were created for peptides which differ in their biochemical characteristics (S1 Table). In a last step, all obtained peptide sequences from *C. salei* and *C. getazi* were used as query and blasted against the UniProtKB database.

The data for this study have been deposited in the European Nucleotide Archive (ENA) at EMBL-EBI under accession number PRJEB42022 (https://www.ebi.ac.uk/ena/browser/view/PRJEB42022). Accession numbers as well as the characteristics of the deposited cDNA sequences (N = 283) are summarized in S2Table.

Signal peptides were identified using SignalP v. 5.0^43^, biochemical characterization of the peptides was done with Expasy/ProtParam^44^, peptide secondary structure prediction with the GOR method^45^, peptide logos were generated with WebLogo (Vers. 2.8.2)^26^, and the visualization of the α-helical peptides as presented in^27^.

#### Insecticidal activity

Cupiennins 3a, 3b, 4a, 6a, 6b, 6d, 7b, 8, and the two-chain CsTx-16a1b1, and CsTx-16c1d1 were synthesized (all C-terminally amidated) by GeneCust, (Dulange, Luxembourg). Bioassays using *Drosophila* flies were performed as described elsewhere^25^. Briefly, all peptides were dissolved in 0.1 M ammonium acetate, pH 6.1 and 0.05 µl injected intrathoracically into the flight muscles of female flies (1- to 7-days old). For each peptide, in minimum five different peptide doses were injection into the flies (each n = 20) and also a negative control with only buffer was injected (n = 10). Calculations of the lethal dose (LD_50_) were done with GraphPad PRISM Vers. 6.07 (GraphPad Software, San Diego, CA, USA).

## Supplementary Information

Supplementary Information 1.

Supplementary Information 2.

Supplementary Information 3.

Supplementary Information 4.

Supplementary Information 5.

Supplementary Information 6.

Supplementary Information 7.
