# Supplementary material for: Complex precursor structures of cytolytic cupiennins identified in spider venom gland transcriptomes
Source: Sci Rep. 2021 Feb 17;11:4009. doi: 10.1038/s41598-021-83624-z (PMC7889660; doi:10.1038/s41598-021-83624-z)
Supplement: Supplementary file 1 — Supplementary Information 1. [file 41598_2021_83624_MOESM1_ESM.pdf]

Complex precursor structures of cytolytic cupiennins identified in spider venom gland transcriptomes

Nature Scientific Reports
Lucia Kuhn-Nentwig
Institute of Ecology and Evolution, University of Bern, Baltzerstrasse 6, 3012 Bern, Switzerland
lucia.kuhn@iee.unibe.ch

Supporting information S1 Fig.pdf

Graphical overview on the composition of the transcript

- Supporting information 1A: A1,2 families
- Supporting information 1B: B1,2,3,4,5 families
- Supporting information 1C: C1 family
- Supporting information 1D: C2 family
- Supporting information 1E: D1,2 families

Supporting information 1A

Transcript A1,2 families

GOR secondary structure prediction method version IV,
J. Garnier, J.-F. Gibrat, B. Robson, Methods in Enzymology,
R.F. Doolittle Ed., 266, 540-553, (1996)

Secondary structure
(hh) Alpha helix (%)
(ee) Extended strand
(cc) Random coil

[ ] read counts
Cu x verified by former publication
Cu x verified by former publication of truncated variant
Cu x predicted
Cu x sequence present in more than 1 transcript family
sequence order and possible short cuts
cDNA data: supplementary material 2.pdf

Signal peptide
[615]

MNYLPIICLLLVALASSTTSS

Propeptide
[A1 1018][A2 129]

A1
SDVPQSELQRKESINSIEDETQRALELTQGNEEETRSENEIDEEDER
ccccccchhhhhccccchhhhhhhhhhhcccchhhhhhhhhccccee 53.2%

A2
SDVPQSELQRKESINSIEDETQRALELTQGNEEETRTENEIDEEDER
ccccccchhhhhccccchhhhhhhhhhhcccchhhhhhhhhccccee 53.2%

Cu 1a
[A1_721]
[A2_73]

Cu 1e
[8]

GFGALFKFLAKKVAKTVAKQAAKQGAKYVVNKQMEG
ccccchhhhhhhhhhhhhhhhhhhceeeeeeeccee 52.8%

1 A1: Cu 1a mainly exhibits Y[28] as (TAC)
2 A2: Cu 1a exhibits Y[28] as (TAT)

GFGALFKFLAKKVAKTVAKQAAKQGAKYIANKQTEG
ccccchhhhhhhhhhhhhhhhhhhhhhhhhccceec 69.4%

TAGAAGAAGTGA

3 RSLEGTFAKTQF STOP KK STOP [A1_8]
4 RTLEGTFAKTQF STOP KK STOP [A2_12]

RTENDSDEEDER
RTENEIDEEDER

Cu 4c
[941]

RIENDSEEEDER

GFGMLFKFLAKKVGKQLASHIAKKQLEG
cccceeehhhhhhhhhhhhhhhhcceec 57.1%

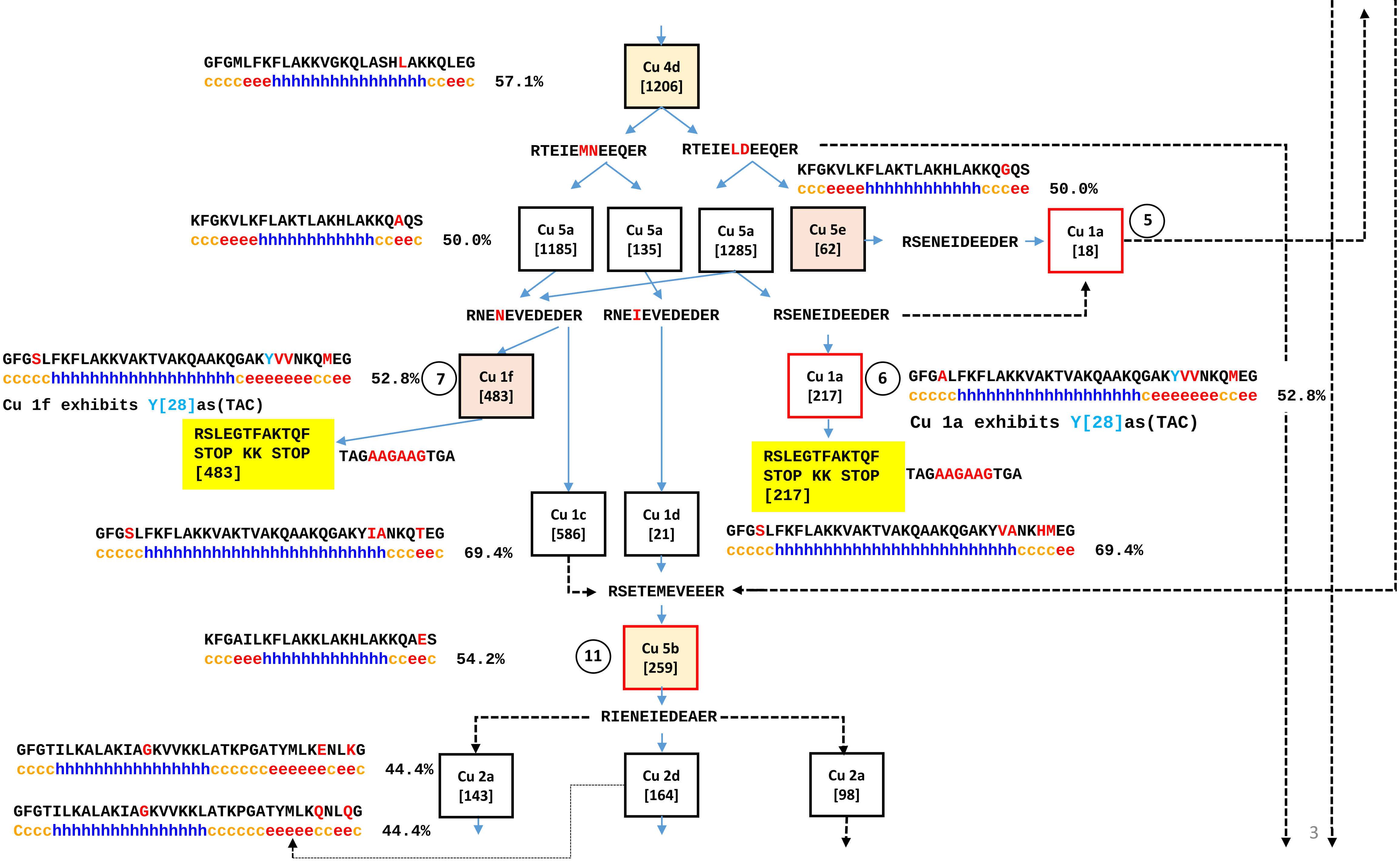
GFGMLFKFLAKKVGKQLASHLAKKQLEG
cccceeehhhhhhhhhhhhhhhcceec 57.1%
Cu 4d
[1206]
RTEIEMNEEQER
RTEIELDEEQER
KFGKVLKFLAKTLAKHLAKKQGQS
ccceeeehhhhhhhhhhhcccee 50.0%
KFGKVLKFLAKTLAKHLAKKQAQS
ccceeeehhhhhhhhhhhcceec 50.0%
Cu 5a
[1185]
Cu 5a
[135]
Cu 5a
[1285]
Cu 5e
[62]
RSENEIDEEDER
Cu 1a
[18]
5
RNENEVEDEDER
RNEIEVEDEDER
RSENEIDEEDER
GFGSLFKFLAKKVAKTVAKQAAKQGAKYVVNKQMEG
cccccchhhhhhhhhhhhhhhhhhhceeeeeeeccee 52.8%
7
Cu 1f
[483]
Cu 1f exhibits Y[28]as(TAC)
RSLEGTFAKTQF
STOP KK STOP
[483]
TAGAAGAAGTGA
Cu 1a
[217]
6
GFGALFKFLAKKVAKTVAKQAAKQGAKYVVNKQMEG
cccccchhhhhhhhhhhhhhhhhhhceeeeeeeccee 52.8%
Cu 1a exhibits Y[28]as(TAC)
RSLEGTFAKTQF
STOP KK STOP
[217]
TAGAAGAAGTGA
GFGSLFKFLAKKVAKTVAKQAAKQGAKYIANKQTEG
cccccchhhhhhhhhhhhhhhhhhhhhhhhhhccceec 69.4%
Cu 1c
[586]
Cu 1d
[21]
GFGSLFKFLAKKVAKTVAKQAAKQGAKYVANKHMEG
cccccchhhhhhhhhhhhhhhhhhhhhhhhhhhccccee 69.4%
RSETEMEVEEER
KFGAILKFLAKKLAKHLAKKQAES
ccceeehhhhhhhhhhhhhcceec 54.2%
11
Cu 5b
[259]
RIENEIEDEAER
GFGTILKALAKIAGKVVKKLATKPGATYMLKENLKG
cccchhhhhhhhhhhhhhhhcccccceeeeeeceec 44.4%
Cu 2a
[143]
Cu 2d
[164]
Cu 2a
[98]
GFGTILKALAKIAGKVVKKLATKPGATYMLKQNLQG
Ccccchhhhhhhhhhhhhhhhcccccceeeeeecceec 44.4%

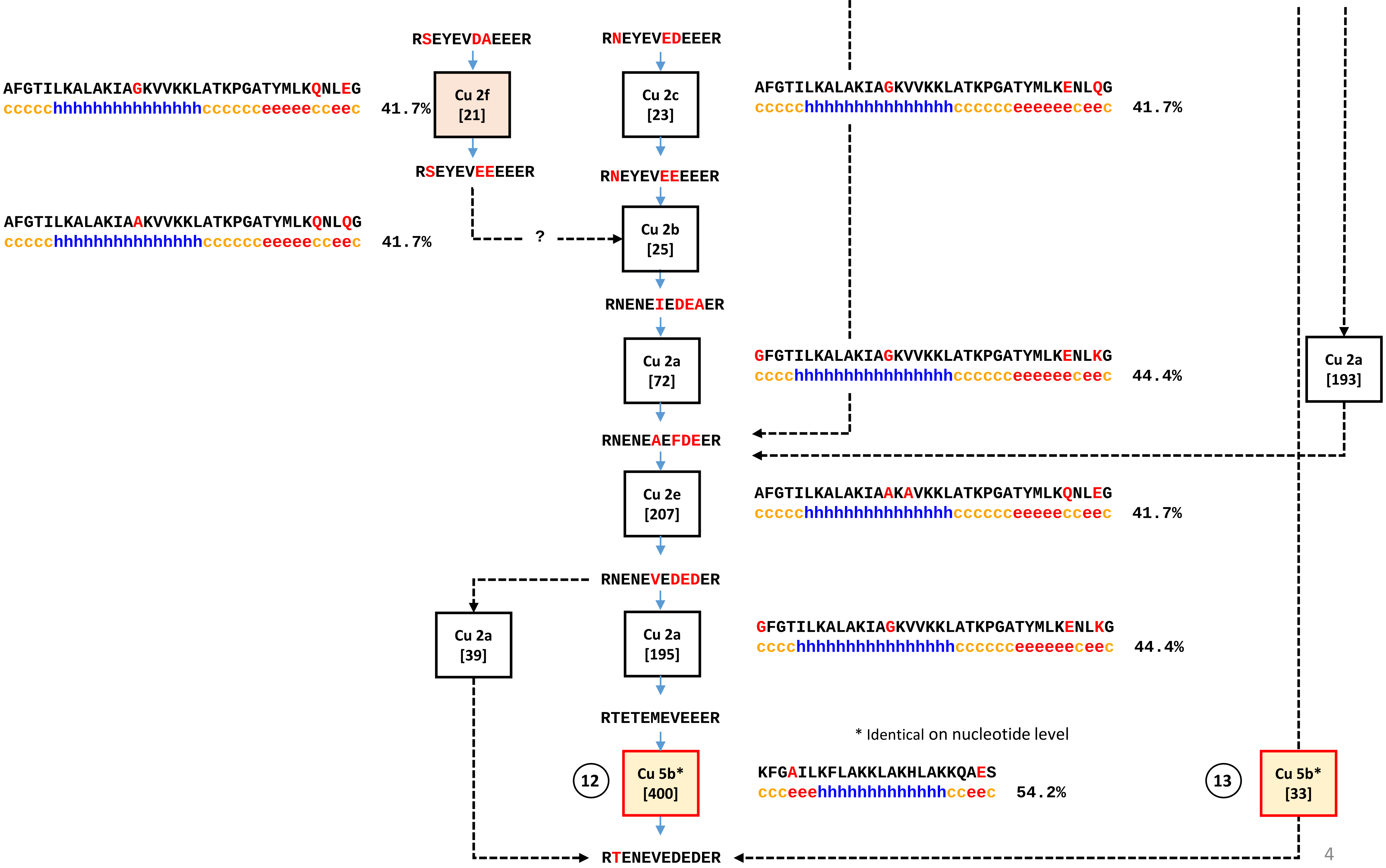
RSEYEVDAEEER
Cu 2f
[21]
RSEYEVEEEEER
AFGTILKALAKIAGKVVKKLATKPGATYMLKQNLEG
ccccchhhhhhhhhhhhhhccccccceeeeeeccceec
41.7%
AFGTILKALAKIAAKVVKKLATKPGATYMLKQNLQG
ccccchhhhhhhhhhhhhhccccccceeeeeeccceec
41.7%
?
RNEYEVEDEEER
Cu 2c
[23]
RNEYEVEEEEER
Cu 2b
[25]
RNENEIEDEAER
Cu 2a
[72]
RNENEAEFDEER
Cu 2e
[207]
RNENEVEDEDER
Cu 2a
[195]
Cu 2a
[39]
RTETEMEVEEER
12
Cu 5b*
[400]
RTENEVEDEDER
AFGTILKALAKIAGKVVKKLATKPGATYMLKENLQG
ccccchhhhhhhhhhhhhhccccccceeeeeeccceec
41.7%
GFGTILKALAKIAGKVVKKLATKPGATYMLKENLKG
cccchhhhhhhhhhhhhhhhcccccceeeeeeceec
44.4%
AFGTILKALAKIAAKAVKKLATKPGATYMLKQNLEG
ccccchhhhhhhhhhhhhhcccccceeeeeccceec
41.7%
GFGTILKALAKIAGKVVKKLATKPGATYMLKENLKG
cccchhhhhhhhhhhhhhhhcccccceeeeeeceec
44.4%
* Identical on nucleotide level
KFGAILKFLAKKLAKHLAKKQAES
ccceeehhhhhhhhhhhhhcceec
54.2%
Cu 2a
[193]
13
Cu 5b*
[33]

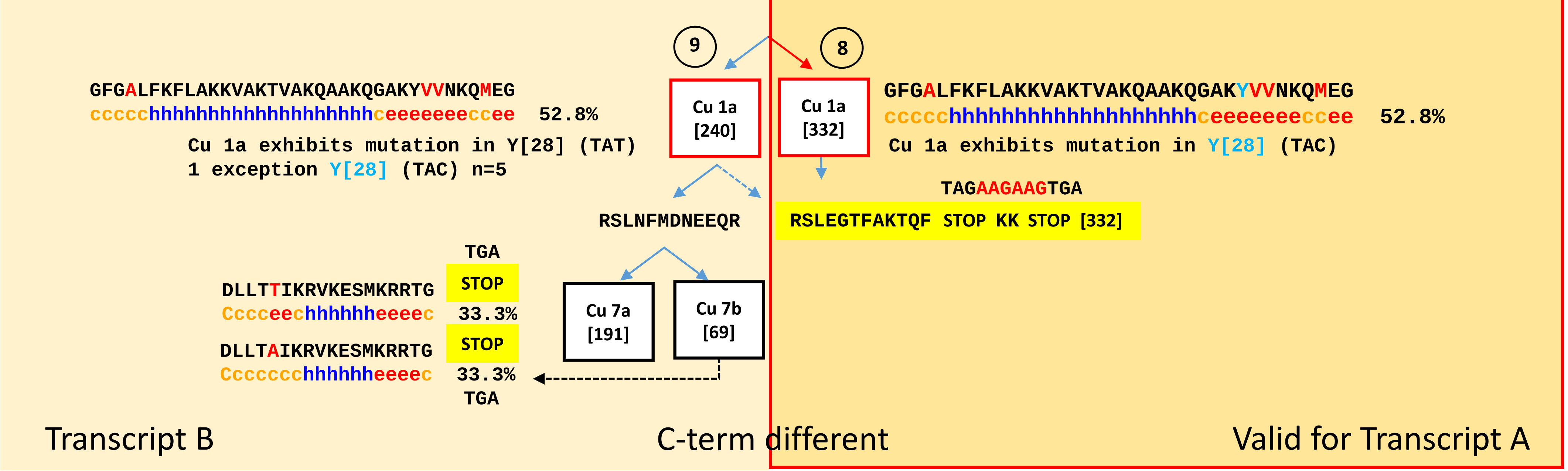

9
8
Cu 1a [240]
Cu 1a [332]
GFGALFKFLAKKVAKTVAKQAAKQGAKYVVNKQMEG
cccccchhhhhhhhhhhhhhhhhhhceeeeeeeccee 52.8%
Cu 1a exhibits mutation in Y[28] (TAT)
1 exception Y[28] (TAC) n=5
GFGALFKFLAKKVAKTVAKQAAKQGAKYVVNKQMEG
cccccchhhhhhhhhhhhhhhhhhhceeeeeeeccee 52.8%
Cu 1a exhibits mutation in Y[28] (TAC)
TAGAAGAAGTGA
RSLEGTFAKTQF STOP KK STOP [332]
RSLNFMDNEEQR
TGA
DLLTTIKRVKESMKRRTG
STOP
Ccccceechhhhhheeeec
33.3%
DLLTAIKRVKESMKRRTG
STOP
Cccccccchhhhhheeeec
33.3%
TGA
Cu 7a [191]
Cu 7b [69]
Transcript B
C-term different
Valid for Transcript A

Supporting information 1B

Transcript B1,2,3,4,5 families

GOR secondary structure prediction method version IV,
J. Garnier, J.-F. Gibrat, B. Robson, Methods in Enzymology,
R.F. Doolittle Ed., 266, 540-553, (1996)

Secondary structure
(hh) Alpha helix (%)
(ee) Extended strand
(cc) Random coil

[ ] read counts
Cu x verified by former publication
Cu x verified by former publication of truncated variant
Cu x predicted

Cu x sequence present in more than 1 transcript family
sequence order and possible short cuts

cDNA data: Supplementary material 2.pdf

*Only such SPs are counted, which contain part of the N-terminal propeptide, because SP is shared with transcript C

Signal peptide
[2814]*

MNYLQVICLLLVALACSTTSS

Propeptide
[B1 604][B2 45][B3 41][B4 2115][B5 9]

B1
SDVPQNELQREENSIEDETLRGLELMQENAEETRIENEIDEEDER
cccccccccchhhhhhhhhhhhhhhhhhhhhhhhhhhhhhhhhhcccccee 68.2%

B2
SDVPQNELQREENSIEDETLRGLELMQENAEETRTENEIDEEDER
cccccccccchhhhhhhhhhhhhhhhhhhhhhhhhhhhhhhhhhcccccee 68.2%

B3
SDVPQNELQREENSIEDETLRGLELMQENAEETRNENESDEEDER
cccccccccchhhhhhhhhhhhhhhhhhhhhhhhhhhhhhhcccccceeec 61.4%

B5
SDVPQNELQREENSIEDETLRGLELMQENAEETRTETDNDVEVER
cccccccccchhhhhhhhhhhhhhhhhhhhhhhhhhhhhcccccccceeeec 56.8%

B4
SDVPQNELQREENSIEDETLRGLELMQENAEETRSENEIDEEDQR
cccccccccchhhhhhhhhhhhhhhhhhhhhhhhhhhhhhhhhhcccccee 68.2%

Cu 8a
[2047]

IWRMPKKVLKLLAAFRGKEG
ccccccchhhhhhhcceec 40.0%

RIENEIDEEDER RTENEIDEEDER

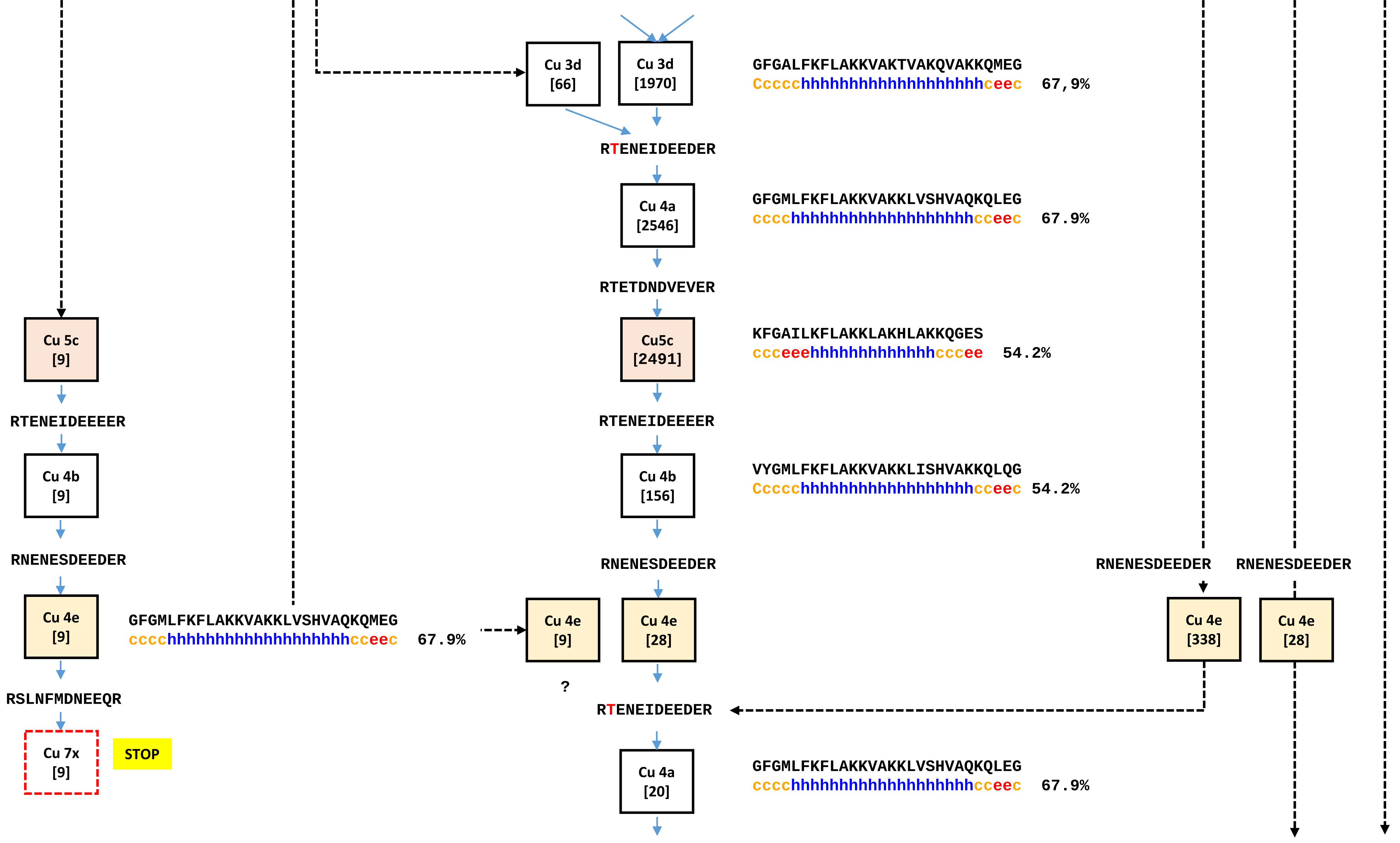
Cu 3d [66]
Cu 3d [1970]
GFGALFKFLAKKVAKTVAKQVAKKQMEG
Cccccchhhhhhhhhhhhhhhhhhhceec 67,9%
RTENEIDEEDER
Cu 4a [2546]
GFGMLFKFLAKKVAKKLVSHVAQKQLEG
cccchhhhhhhhhhhhhhhhhhhcceec 67.9%
RTETDNDVEVER
Cu5c [2491]
KFGAILKFLAKKLAKHLAKKQGES
ccceeehhhhhhhhhhhhhccccee 54.2%
RTENEIDEEEER
Cu 4b [156]
VYGMLFKFLAKKVAKKLISHVAKKQLQG
Ccccchhhhhhhhhhhhhhhhhcceec 54.2%
RNENESDEEDER
Cu 5c [9]
RTENEIDEEEER
Cu 4b [9]
RNENESDEEDER
Cu 4e [9]
GFGMLFKFLAKKVAKKLVSHVAQKQMEG
cccchhhhhhhhhhhhhhhhhhcceec 67.9%
RSLNFMDNEEQR
Cu 7x [9]
STOP
Cu 4e [9]
?
Cu 4e [28]
RNENESDEEDER
RNENESDEEDER
Cu 4e [338]
Cu 4e [28]
RTENEIDEEDER
Cu 4a [20]
GFGMLFKFLAKKVAKKLVSHVAQKQLEG
cccchhhhhhhhhhhhhhhhhhhcceec 67.9%

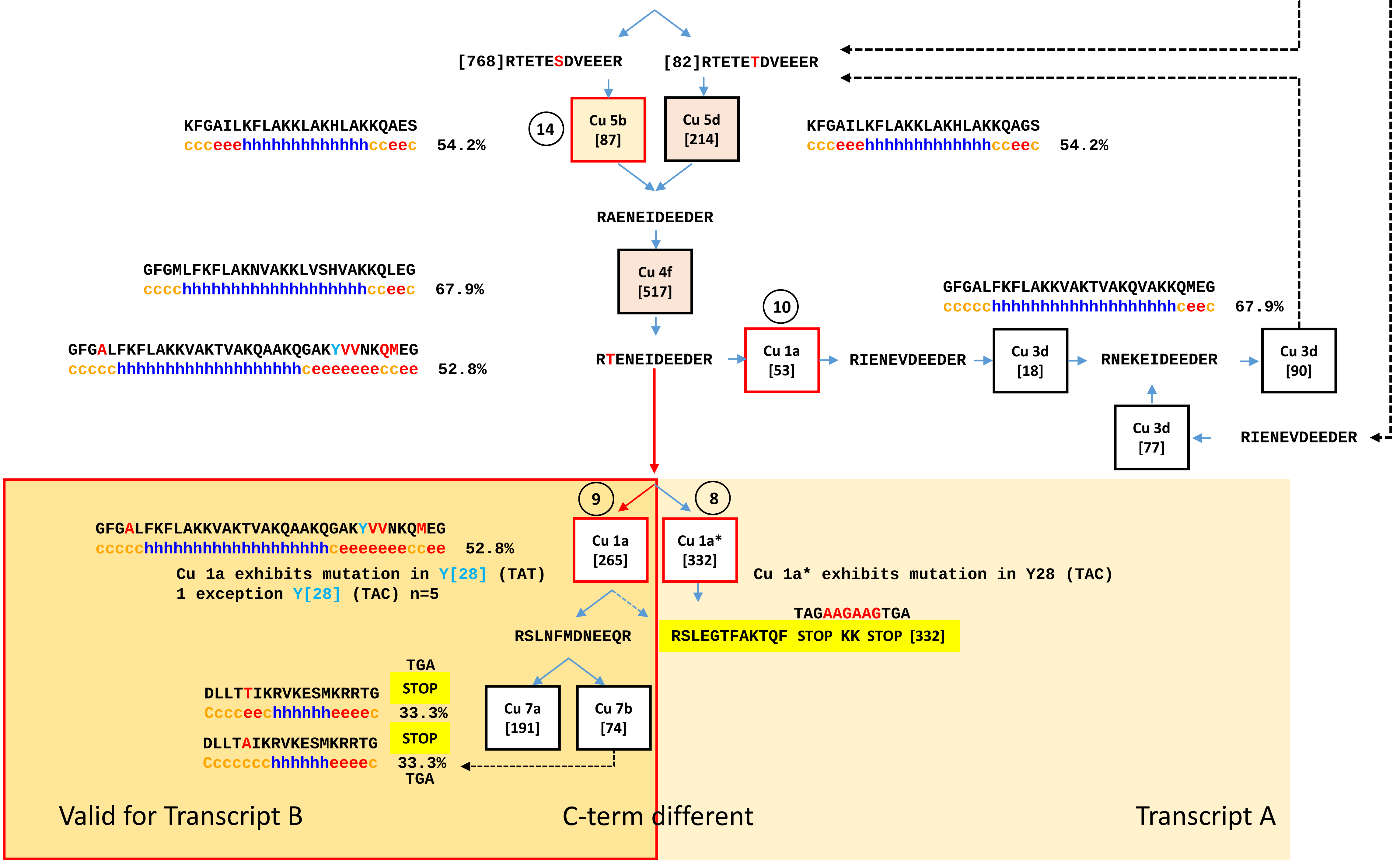

[768]RTETESDVEEER
[82]RTETETDVEEER
KFGAILKFLAKKLAKHLAKKQAES
ccceeehhhhhhhhhhhhhcceec
54.2%
14
Cu 5b [87]
Cu 5d [214]
KFGAILKFLAKKLAKHLAKKQAGS
ccceeehhhhhhhhhhhhhcceec
54.2%
RAENEIDEEDER
GFGMLFKFLAKNVAKKLVSHVAKKQLEG
cccchhhhhhhhhhhhhhhhhhhcceec
67.9%
Cu 4f [517]
GFGALFKFLAKKVAKTVAKQVAKKQMEG
cccchhhhhhhhhhhhhhhhhhhhceec
67.9%
10
GFGALFKFLAKKVAKTVAKQAAKQGAKYVVNKQMEG
ccccchhhhhhhhhhhhhhhhhhhceeeeeeeccee
52.8%
RTENEIDEEDER
Cu 1a [53]
RIENEVDEEDER
Cu 3d [18]
RNEKEIDEEDER
Cu 3d [90]
Cu 3d [77]
RIENEVDEEDER
9
8
GFGALFKFLAKKVAKTVAKQAAKQGAKYVVNKQMEG
ccccchhhhhhhhhhhhhhhhhhhceeeeeeeccee
52.8%
Cu 1a [265]
Cu 1a* [332]
Cu 1a exhibits mutation in Y[28] (TAT)
1 exception Y[28] (TAC) n=5
Cu 1a* exhibits mutation in Y28 (TAC)
TAGAAGAAGTGA
RSLEGTFAKTQF STOP KK STOP [332]
RSLNFMDNEEQR
TGA
DLLTTIKRVKESMKRRTG STOP
Cccceechhhhhheeeec
33.3%
Cu 7a [191]
Cu 7b [74]
DLLTAIKRVKESMKRRTG STOP
Ccccccchhhhhheeeec
33.3%
TGA
Valid for Transcript B
C-term different
Transcript A

Supporting information 1C

Transcript C1 family

Signal peptide
[5270]*

MNYLQVICLLLVALACSTTSS

↓

Propeptide
[5310]

SDVPQKELQEQREESINSIEDETQRTLELKQRTEEEMRTENEVDEEDER
ccccccchhhhhhhhhchhhhhhhhhhhhhhhhhhhhhhhhccccccee 68.8%

↓

GFGSLFKFLGKKLLKTVAKQAAKKQMEG
ccccchhhhhhhhhhhhhhhhhhhceec 67.9%

Cu 3a
[3057]

↓

RSSTDSDNVEGR

↓

GOR secondary structure prediction method version IV, J. Garnier, J.-F. Gibrat, B. Robson, Methods in Enzymology, R.F. Doolittle Ed., 266, 540-553, (1996)

Secondary structure

(hh) Alpha helix (%)

(ee) Extended strand

(cc) Random coil

[ ]	read counts
**Cu x**	verified by former publication
**Cu x**	verified by former publication of truncated variant
**Cu x**	predicted
→	sequence order and possible short cuts
- - -►	

*Only such SPs are counted, which contain part of the N-terminal propeptide, because SP is shared with transcript B

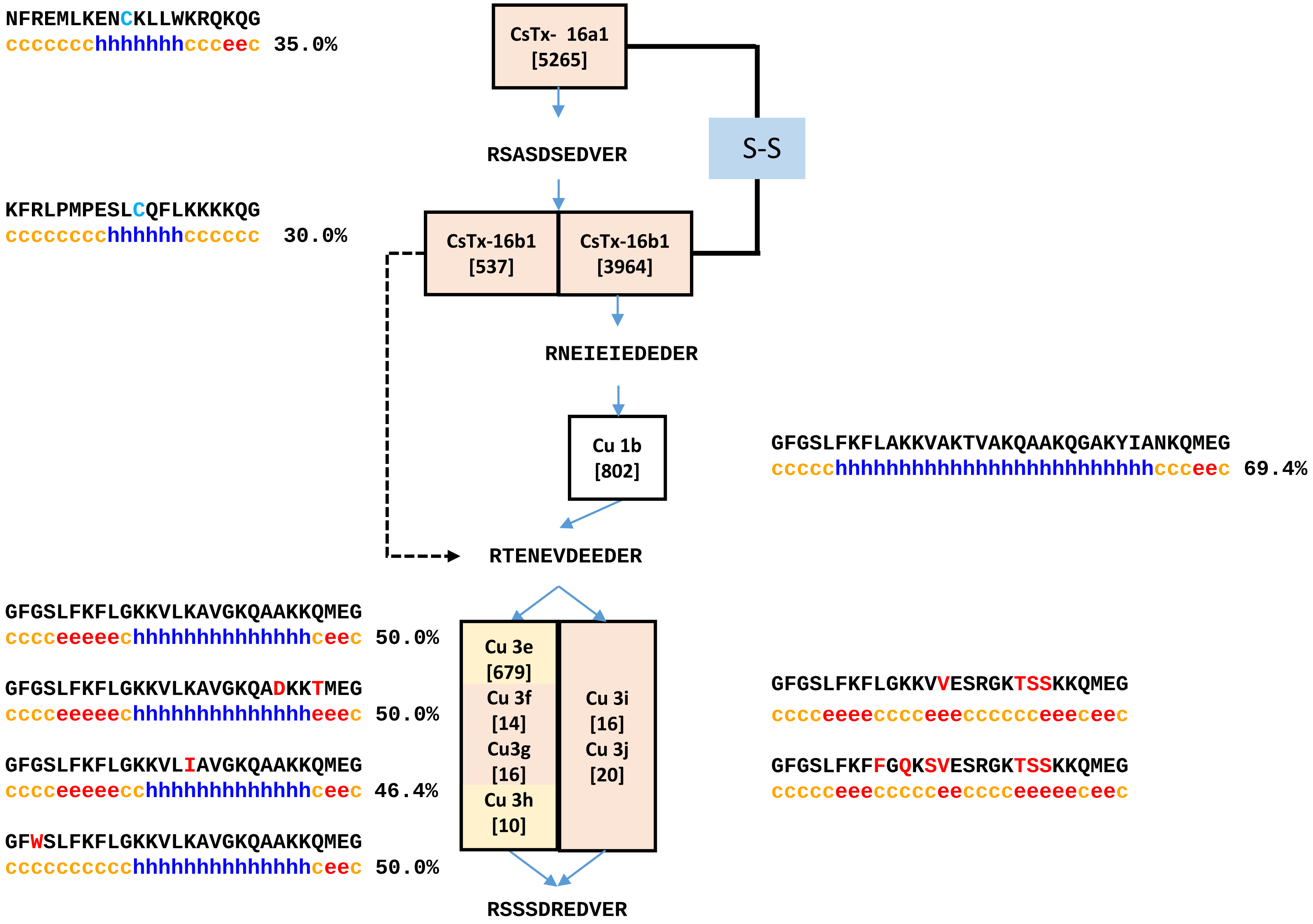
NFREMLKENCKLLWKRQKQG
cccccchhhhhhhccceec 35.0%
CsTx- 16a1
[5265]
RSASDSEDVER
S-S
KFRLPMPESLCQFLKKKKQG
ccccccchhhhhhcccccc 30.0%
CsTx-16b1
[537]
CsTx-16b1
[3964]
RNEIEIEDEDER
Cu 1b
[802]
GFGSLFKFLAKKVAKTVAKQAAKQGAKYIANKQMEG
cccchhhhhhhhhhhhhhhhhhhhhhhhhhhhccceec 69.4%
RTENEVDEEDER
GFGSLFKFLGKKVLKAVGKQAAKKQMEG
cccceeeeechhhhhhhhhhhhhhceec 50.0%
GFGSLFKFLGKKVLKAVGKQADKKTMEG
cccceeeeechhhhhhhhhhhhheeec 50.0%
GFGSLFKFLGKKVLIAVGKQAAKKQMEG
cccceeeeecchhhhhhhhhhhhceec 46.4%
GFWSLFKFLGKKVLKAVGKQAAKKQMEG
ccccccccchhhhhhhhhhhhhhceec 50.0%
Cu 3e
[679]
Cu 3f
[14]
Cu3g
[16]
Cu 3h
[10]
Cu 3i
[16]
Cu 3j
[20]
GFGSLFKFLGKKVVESRGKTSSKKQMEG
ccccceeeeccccceeeccccccceeeceec
GFGSLFKFFGQKSVESRGKTSSKKQMEG
cccccceeeccccceeecccceeeeeceec
RSSSDREDVER

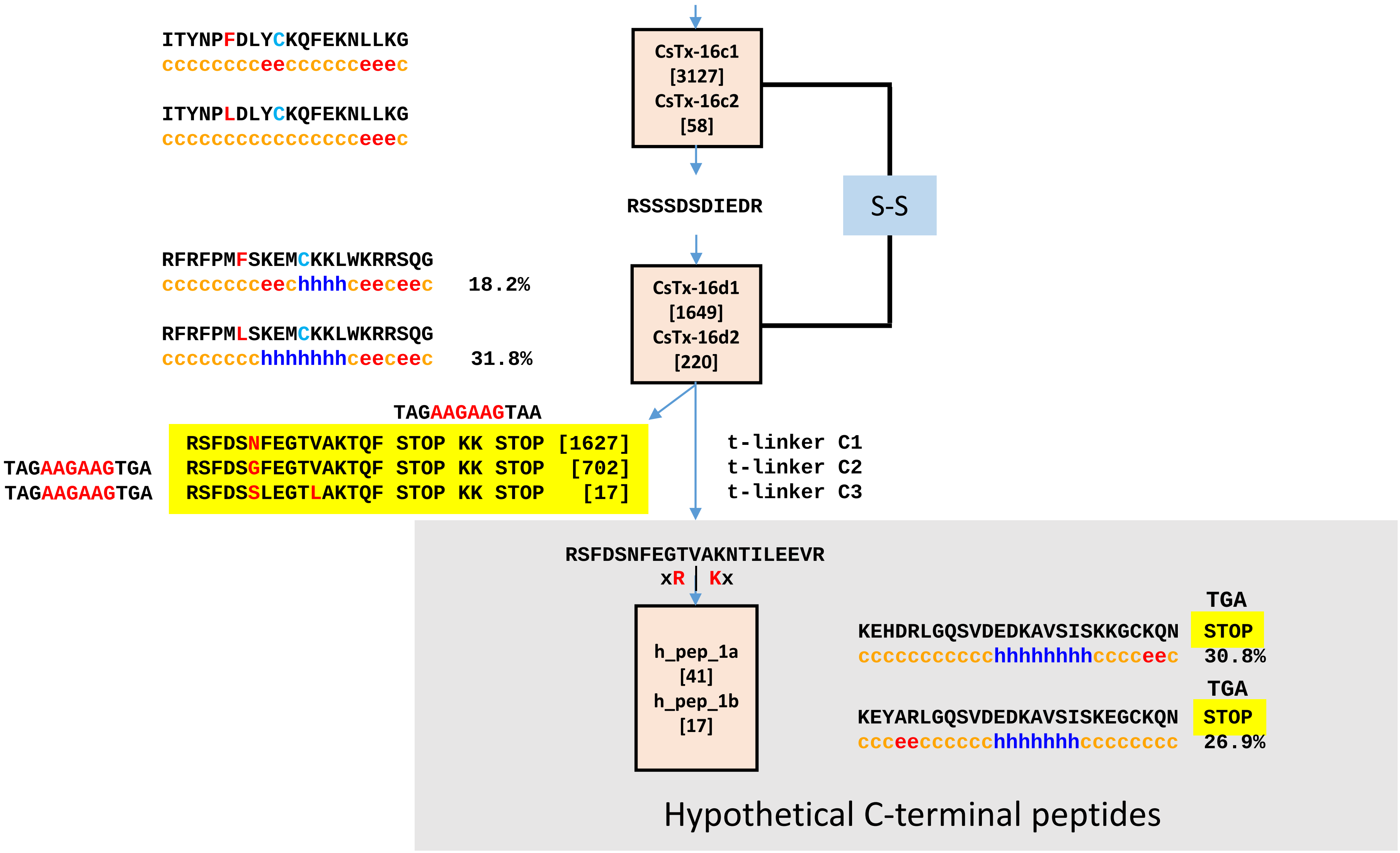
ITYNPFDLYCKQFEKNLLKG
ccccccccceeccccccceeec
ITYNPLDLYCKQFEKNLLKG
ccccccccccccccccceeec
CsTx-16c1
[3127]
CsTx-16c2
[58]
RSSSDSDIEDR
S-S
RFRFPMFSKEMCKKLWKRRSQG
ccccccccceechhhhceeceec
18.2%
RFRFPMLSKEMCKKLWKRRSQG
cccccccchhhhhhceeceec
31.8%
CsTx-16d1
[1649]
CsTx-16d2
[220]
TAGAAGAAGTAA
RSFDSNFEGTVAKTQF STOP KK STOP [1627]
TAGAAGAAGTGA
RSFDSGFEGTVAKTQF STOP KK STOP [702]
TAGAAGAAGTGA
RSFDSSLEGTLAKTQF STOP KK STOP [17]
t-linker C1
t-linker C2
t-linker C3
RSFDSNFEGTVAKNTILEEVR
xR | Kx
h_pep_1a
[41]
h_pep_1b
[17]
TGA
KEHDRLGQSVDEDKAVSISKKGCKQN
STOP
cccccccccccchhhhhhhcccceec
30.8%
TGA
KEYARLGQSVDEDKAVSISKEGCKQN
STOP
ccceecccccchhhhhhcccccccc
26.9%
Hypothetical C-terminal peptides

Supporting information 1D

Transcript C2 family

Signal peptide
[109]*

MNYLQVICLLIVALACSTTSS

Propeptide
[326]

SDVPQKELQEQREESSSDSDHVEGR
cccccchhhhhhccccccceeeec 28.0%

CsTx-16e
[240]

DLTETYNNLCIVYEKKKMQG
ccccccccceeeeeeccccee

RSASDSEDVER

S-S

CsTx-16f
[209]

IFRNPIQKTLCKLMKKKLLQG
cccccccchhhhhhhccceec 33.3%

RNENEVEDEDESR

GOR secondary structure prediction method version IV, J. Garnier, J.-F. Gibrat, B. Robson, Methods in Enzymology, R.F. Doolittle Ed., 266, 540-553, (1996)

Secondary structure

(hh) Alpha helix (%)
(ee) Extended strand
(cc) Random coil

[ ] read counts
Cu x verified by former publication
Cu x verified by former publication of truncated variant
Cu x predicted

→ sequence order

*SP differs from SP of transcript B and transcript C1 only in 1 amino acid residue

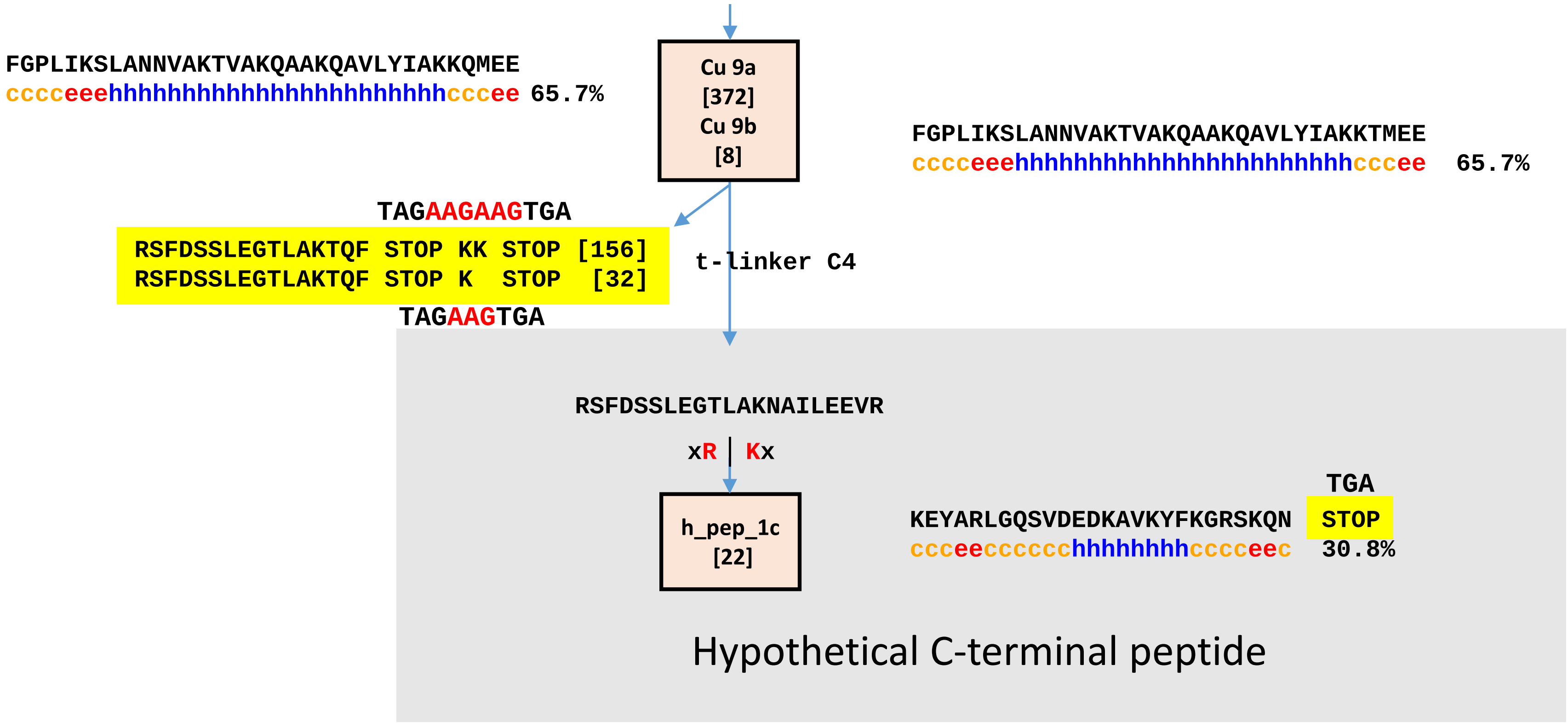
FGPLIKSLANNVAKTVAKQAAKQAVLYIAKKQMEE
cccceeehhhhhhhhhhhhhhhhhhhhhhhcccee 65.7%
Cu 9a
[372]
Cu 9b
[8]
FGPLIKSLANNVAKTVAKQAAKQAVLYIAKKTMEE
cccceeehhhhhhhhhhhhhhhhhhhhhhhcccee 65.7%
TAGAAGAAGTGA
RSFDSSLEGTLAKTQF STOP KK STOP [156]
RSFDSSLEGTLAKTQF STOP K STOP [32]
TAGAAGTGA
t-linker C4
RSFDSSLEGTLAKNAILEEVR
xR | Kx
h_pep_1c
[22]
TGA
KEYARLGQSVDEDKAVKYFKGRSKQN STOP
ccceeccccccchhhhhhhccccceec 30.8%
Hypothetical C-terminal peptide

Supporting information 1E

Transcript D1,2 families

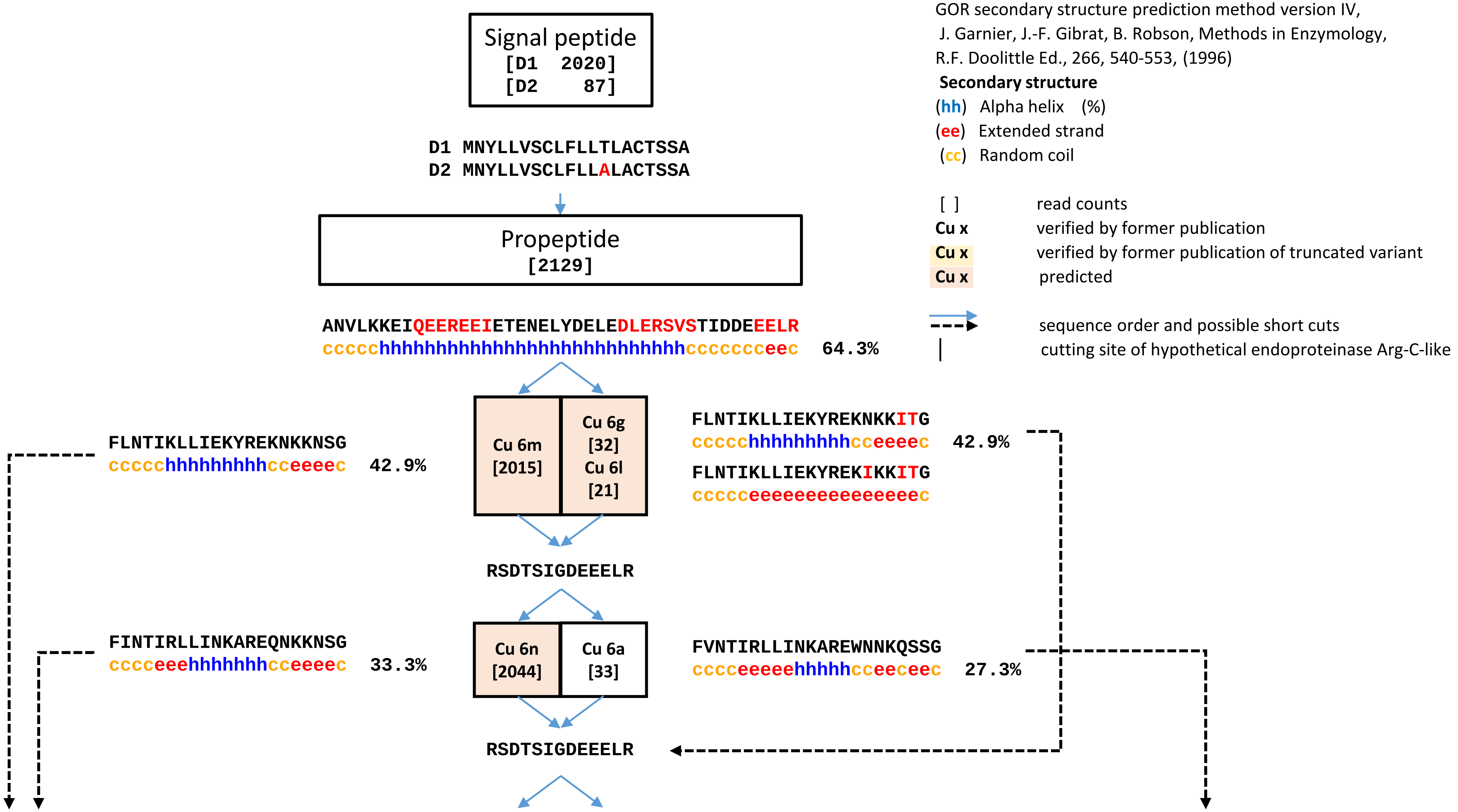

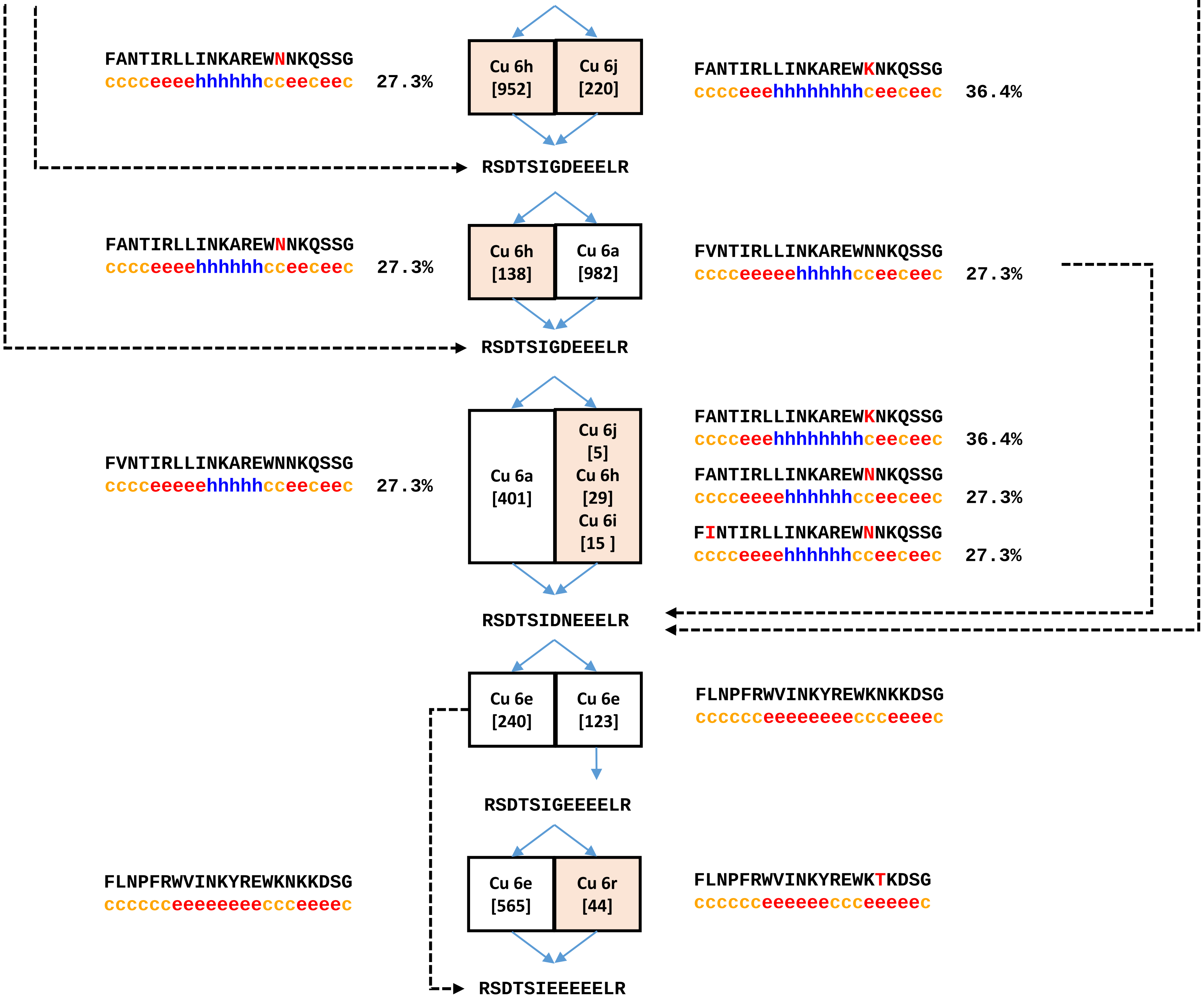
FANTIRLLINKAREWNNKQSSG
ccccceeeehhhhhhccceeceec
27.3%
Cu 6h
[952]
Cu 6j
[220]
FANTIRLLINKAREWKNKQSSG
ccccceeehhhhhhhceeceec
36.4%
RSDTSIGDEEELR
FANTIRLLINKAREWNNKQSSG
ccccceeeehhhhhhccceeceec
27.3%
Cu 6h
[138]
Cu 6a
[982]
FVNTIRLLINKAREWNNKQSSG
ccccceeeeehhhhhccceeceec
27.3%
RSDTSIGDEEELR
FVNTIRLLINKAREWNNKQSSG
ccccceeeeehhhhhccceeceec
27.3%
Cu 6a
[401]
Cu 6j
[5]
Cu 6h
[29]
Cu 6i
[15]
FANTIRLLINKAREWKNKQSSG
ccccceeehhhhhhhceeceec
36.4%
FANTIRLLINKAREWNNKQSSG
ccccceeeehhhhhhccceeceec
27.3%
FINTIRLLINKAREWNNKQSSG
ccccceeeehhhhhhccceeceec
27.3%
RSDTSIDNEEELR
Cu 6e
[240]
Cu 6e
[123]
FLNPFRWVINKYREWKNKKDSG
cccccceeeeeeeecccceeeec
RSDTSIGEEEELR
FLNPFRWVINKYREWKNKKDSG
cccccceeeeeeeecccceeeec
Cu 6e
[565]
Cu 6r
[44]
FLNPFRWVINKYREWKTKDSG
cccccceeeeeecccceeeeec
RSDTSIEEEEELR

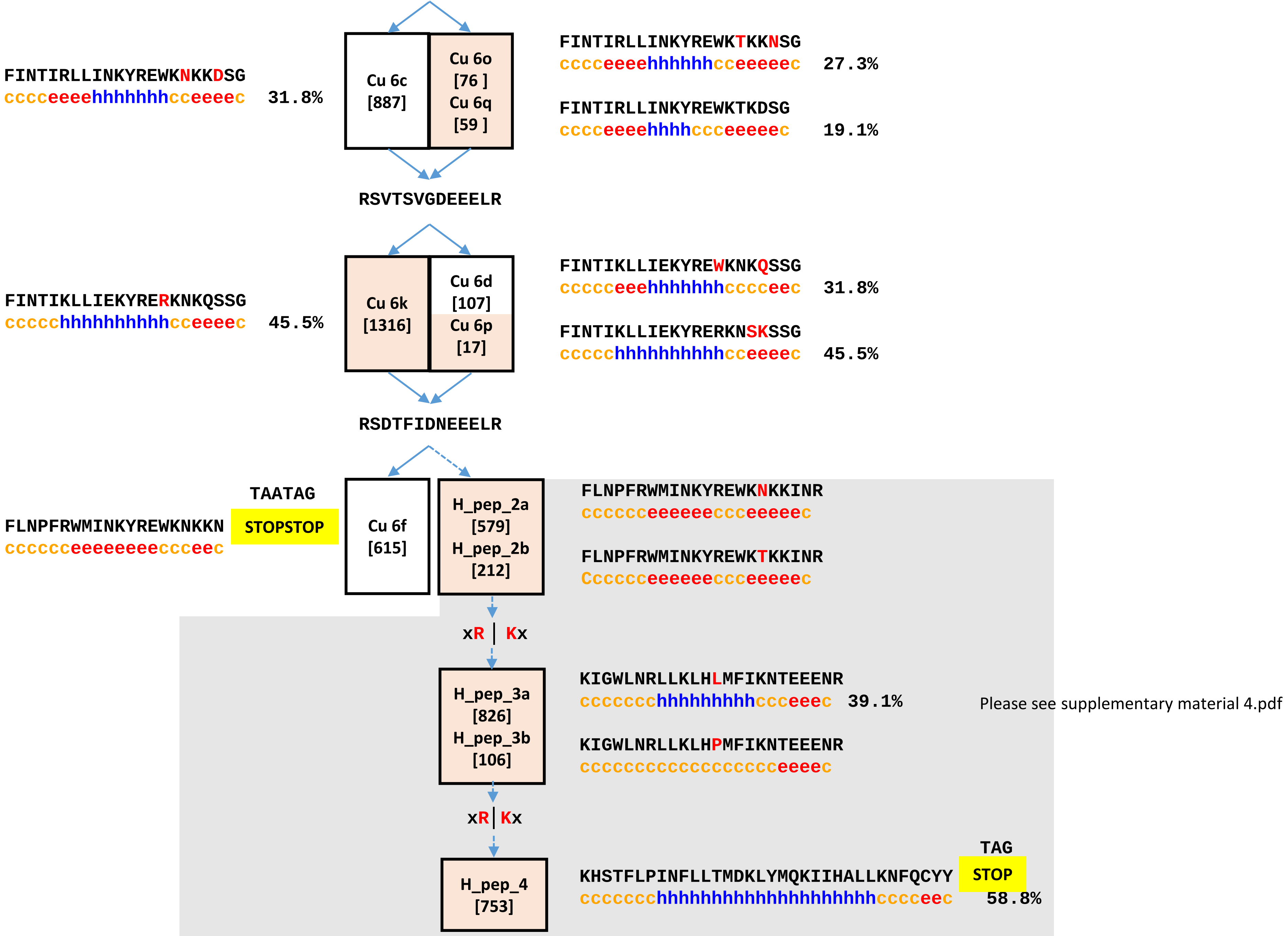

Hypothetical C-terminal peptides
