# Supplementary material for: Complex precursor structures of cytolytic cupiennins identified in spider venom gland transcriptomes
Source: Sci Rep. 2021 Feb 17;11:4009. doi: 10.1038/s41598-021-83624-z (PMC7889660; doi:10.1038/s41598-021-83624-z)
Supplement: Supplementary file 3 — Supplementary Information 3. [file 41598_2021_83624_MOESM3_ESM.pdf]

Complex precursor structures of cytolytic cupiennins identified in spider venom gland transcriptomes

Nature Scientific Reports
Lucia Kuhn-Nentwig
Institute of Ecology and Evolution, University of Bern, Baltzerstrasse 6, 3012 Bern, Switzerland
lucia.kuhn@iee.unibe.ch

Supporting information S2 Fig.pdf
Supporting information 2A: Nucleotide sequence analysis of Cu 1a / Cu 1f in transcript families A1, A2, and B, page 2
Supporting information 2B: Nucleotide sequence analysis of Cu 5b in transcript families A1, A2, and B, page 3

Supporting information 2A

Nucleotide sequence analysis of Cu 1a / Cu 1f in transcript families A1, A2, and B

Numbers in circles are corresponding to numbers in circles in supporting information 1.pdf (first part: composition of transcript A1, A2, and B families)

```
(1) Transcript A1 family        Cupiennin 1a variant TAC
>Contig_Spider_Gland_98_7864    GGTTTTGGTGCGTTATTCAAATTTTTGGCCAAAAAAGTGGCGAAAACAGTGGCAAAACAAGCAGCAAAACAAGGTGCAAAATACGTAGTAAACAAACAAATGGAAGGAAGG
>Contig_Spider_Gland_98_19670   GGTTTTGGTGCGTTATTCAAATTTTTGGCCAAAAAAGTGGCGAAAACAGTGGCAAAACAAGCAGCAAAACAAGGTGCAAAATACGTAGTAAACAAACAAATGGAAGGAAGA
>Contig_Spider_Gland_98_28038   GGTTTTGGTGCGTTATTCAAATTTTTGGCTAAAAAAGTGGCGAAAACAGTGGCAAAACAAGCAGCAAAACAAGGAGCAAAATACGTAGTAAACAAACAAATGGAAGGAAGG
(5) Transcript A1 family        Cupiennin 1a variant TAC
>Contig_Spider_Gland_98_8361    GGTTTTGGTGCGTTATTCAAATTTTTGGCTAAAAAAGTGGCGAAAACAGTGGCAAAACAAGCAGCAAAACAAGGAGCAAAATACGTAGTAAACAAACAAATGGAAGGAAGG
                                 G  F  G  A  L  F  K  F  L  A  K  K  V  A  K  T  V  A  K  Q  A  A  K  Q  G  A  K  Y  V  V  N  K  Q  M  E  G  R
```

```
(3) Transcript A1 family        Cupiennin 1a variant TAC                                                                                 t-linker
>Contig_Spider_Gland_98_995     GGTTTTGGTGCGTTATTCAAATTTTTGGCCAAAAAAGTGGCGAAAACAGTGGCAAAACAAGCAGCAAAACAAGGAGCAAAATACGTAGTAAACAAACAAATGGAAGGAAGGAGTCTGGAAGGAACTTTTGCAAAAACGCAATTCTAGAAGAAGTGA
>Contig_Spider_Gland_98_629     GGTTTTGGAGCCTTATTCAAATTTTTAGCAAAAAAAGTGGCAAAAACAGTGGCAAAACAAGCAGCAAAACAAGGAGCAAAATACGTAGTAAACAAACAAATGGAAGGAAGGAGTCTGGAAGGAACTTTTGCAAAAACGCAATTCTAGAAGAAGTGA
(7) Transcript A1 family        Cupiennin 1f variant TAC                                                                                 t-linker
>Contig_Spider_Gland_98_1322    GGATTTGGATCCTTATTCAAATTTTTGGCAAAAAAAGTGGCAAAAACAGTGGCAAAACAAGCAGCAAAACAAGGAGCAAAATACGTAGTAAACAAACAAATGGAAGGAAGGAGTCTGGAAGGAACTTTTGCAAAAACGCAATTCTAGAAGAAGTGA
>Contig_Spider_Gland_98_2809    GGATTTGGATCCTTATTCAAATTTTTGGCAAAAAAAGTGGCAAAAACAGTGGCAAAACAAGCAGCGAAACAAGGAGCAAAATACGTAGTAAACAAACAAATGGAAGGAAGGAGTCTGGAAGGAACTTTTGCAAAAACGCAATTCTAGAAGAAGTGA
(8) Transcript A1 family        Cupiennin 1a variant TAC                                                                                 t-linker
>Contig_Spider_Gland_98_824     GGTTTGGAGCCTTATTCAAATTTTTAGCAAAAAAAGTGGCAAAAACAGTGGCAAAACAAGCAGCAAAACAAGGAGCAAAATACGTAGTAAACAAACAAATGGAAGGAAGGAGTCTGGAAGGAACTTTTGCAAAAACGCAATTCTAGAAGAAGTGA
>Contig_Spider_Gland_98_283     GGATTTGGAGCCTTATTCAAATTTTTAGCAAAAAAAGTGGCAAAAACAGTGGCAAAACAAGCAGCAAAACAAGGAGCAAAATACGTAGTAAACAAACAAATGGAAGGAAGGAGTCTGGAAGGAACTTTTGCAAAAACGCAATTCTAGAAGAAGTGA
>Contig_Spider_Gland_98_567     GGATTTGGAGCCTTATTCAAATTTTTAGCAAAAAAAGTGGCAAAAACAGTGGCAAAACAAGCAGCAAAACAAGGAGCAAAATACGTAGTAAACAAACAAAGGGAAGGAAGGAGTCTGGAAGGAACTTTTGCAAAAACGCAATTCTAGAAGAAGTGA
(6) Transcript A1 family        Cupiennin 1a variant TAC                                                                                 t-linker
>Contig_Spider_Gland_98_4220    GGATTTGGAGCCTTATTCAAATTTTTAGCAAAAAAAGTGGCAAAAACAGTGGCAAAACAAGCAGCAAAACAAGGAGCAAAATACGTAGTAAACAAACAAATGGAAGGAAGGAGTCTGGAAGGAACTTTTGCAAAAACGCAATTCTAGAAGAAGTGA
>Contig_Spider_Gland_98_1412    GGTTTTGGTGCGTTATTCAAATTTTTGGCTAAAAAAGTGGCGAAAACAGTGGCAAAACAAGCAGCAAAACAAGGAGCAAAATACGTAGTAAACAAACAAATGGAAGGAAGGAGTCTGGAAGGAACTTTTGCAAAAACGCAATTCTAGAAGAAGTGA
                                 G  F  G  A  L  F  K  F  L  A  K  K  V  A  K  T  V  A  K  Q  A  A  K  Q  G  A  K  Y  V  V  N  K  Q  M  E  G  R  S  L  E  G  T  F  A  K  T  Q  F  *  K  K  *
```

```
(2) Transcript A2 family        Cupiennin 1a variant TAT
>Contig_Spider_Gland_98_7640    GGTTTTGGTGCGTTATTCAAATTTTTGGCCAAAAAAGTGGCGAAAACAGTGGCAAAACAAGCAGCAAAACAAGGAGCAAAATATGTAGTAAACAAACAAATGGAAGGAAGG
(4) Transcript A2 family        Cupiennin 1a variant TAT                                                                                 t-linker
>Contig_Spider_Gland_98_4913    GGTTTTGGTGCGTTATTCAAATTTTTGGCCAAAAAAGTGGCGAAAACAGTGGCAAAACAAGCAGCAAAACAAGGAGCAAAATATGTAGTAAACAAACAAATGGAAGGAAGGACTCTGGAAGGAACTTTTGCAAAAACGCAATTCTAGAAGAAGTGA
                                 G  F  G  A  L  F  K  F  L  A  K  K  V  A  K  T  V  A  K  Q  A  A  K  Q  G  A  K  Y  V  V  N  K  Q  M  E  G  R  S  L  E  G  T  F  A  K  T  Q  F  *  K  K  *
```

```
(10) Transcript B family        Cupiennin 1a variant TAT
>Contig_Spider_Gland_98_8132    GGTTTTGGTGCGTTATTCAAATTTTTGGCAAAAAAAGTGGCGAAAACAGTTGCAAAACAAGCAGCAAAACAAGGAGCAAAATATGTAGTAAACAAACAAATGGAAGGAAGA
(9) Transcript B family         Cupiennin 1a variant TAT                                                                                 linker                                   Cupiennin 7
>Contig_Spider_Gland_98_862     ------------------------------------------------------------------------------AAATATGTAGTAAACAAACAAATGGAAGGAAGGAGCTTAAATTTCATGGACAACGAGGAGCAGAGGGATCTTCTTACG
                                 G  F  G  A  L  F  K  F  L  A  K  K  V  A  K  T  V  A  K  Q  A  A  K  Q  G  A  K  Y  V  V  N  K  Q  M  E  G  R  S  L  N  F  M  D  N  E  E  Q  R  D  L  L  T
```

Supporting information 2B

Nucleotide sequence analysis of Cu 5b in transcript families A1, A2, and B

Numbers in circles are corresponding to numbers in circles in supporting information 1.pdf (first part: composition of transcript A1, A2, and B families)

(11) Transcript A1 family — **Cupiennin 5b variant**

>Contig_Spider_Gland_98_10484 AGATCCGAAACTGAGATGGAAGTGGAAGAGGAGAGAAAATTTGGTGCAATATTGAAATTTTTGGCCAAAAAATTGGCAAAACACTTAGCAAAGAAACAAGCGGAATCTAGAATCGAAAATGAAATCGAGGACGAAGCAGAGAGG

>Contig_Spider_Gland_98_7642 AGATCCGAAACTGAGATGGAAGTGGAAGAGGAGAGAAAATTTGGTGCAATATTGAAATTTTTGGCAAAAAAATTGGCAAAACACTTAGCAAAGAAACAAGCGGAATCTAGAATCGAAAATGAAATCGAGGACGAAGCAGAGAGG

R S E T E M E V E E E R K F G A I L K F L A K K L A K H L A K K Q A E S R I E N E I E D E A E R

(12) Transcript A1 family — **Cupiennin 5b variant**

>Contig_Spider_Gland_98_466 AGAACCGAAACTGAGATGGAAGTCGAAGAGGAGAGAAAATTTGGTGCAATATTGAAATTTTTAGCCAAAAAATTGGCGAAACACTTAGCAAAGAAGCAAGCGGAATCTAGAACCGAAAATGAAGTCGAGGACGAAGATGAGAGG

R T E T E M E V E E E R K F G A I L K F L A K K L A K H L A K K Q A E S R T E N E V E D E D E R

(13) Transcript A1 family — **Cupiennin 5b variant**

>Contig_Spider_Gland_98_1305 AGAACCGAAATTGAGTTGGACGAAGAACAGGAGAGAAAATTTGGTGCAATATTGAAATTTTTAGCCAAAAAATTGGCGAAACACTTAGCAAAGAAGCAAGCGGAATCTAGAACCGAAAATGAAGTCGAGGACGAAGATGAGAGG

R T E I E L D E E Q E R K F G A I L K F L A K K L A K H L A K K Q A E S R T E N E V E D E D E R

(14) Transcript B family — **Cupiennin 5b variant**

>Contig_Spider_Gland_98_2201 AGAACCGAAACTGAAAGCGACGTCGAAGAGGAGAGGAAATTTGGCGCAATTTTGAAATTTTTGGCCAAAAAATTGGCGAAGCACTTAGCAAAGAAACAAGCGGAATCTAGAGCTGAAAATGAGATCGACGAAGAAGATGAGAGG

R T E T E S D V E E E R K F G A I L K F L A K K L A K H L A K K Q A E S R A E N E I D E E D E R

linker | **Cupiennin 5b** | **linker**
