# Supplementary material for: Complex precursor structures of cytolytic cupiennins identified in spider venom gland transcriptomes
Source: Sci Rep. 2021 Feb 17;11:4009. doi: 10.1038/s41598-021-83624-z (PMC7889660; doi:10.1038/s41598-021-83624-z)
Supplement: Supplementary file 5 — Supplementary Information 5. [file 41598_2021_83624_MOESM5_ESM.pdf]

Complex precursor structures of cytolytic cupiennins identified in spider venom gland transcriptomes

Nature Scientific Reports
Lucia Kuhn-Nentwig
Institute of Ecology and Evolution, University of Bern, Baltzerstrasse 6, 3012 Bern, Switzerland
lucia.kuhn@iee.unibe.ch

Supporting information S3 Fig.pdf
Nucleotide sequence analysis of the C-termini of transcript families C1 and C2

Supporting information 3

Nucleotide sequence analysis of the C-termini of transcript families C1 and C2

1 Transcript C1 family — **t-linker**

>Contig_Spider_Gland_98_24

AGGAGCTTTGATTCTAATTTTGAAGGAACTGTTG**CAAAAACA**CAATTCTAGAAGAAGTAA

R S F D S N F E G T V A K T Q F * K K *

1 Transcript C1 family — **t-linker** — insertion of A ↓ — **PQM-motif** — **h_pep_1a**

>Contig_Spider_Gland_98_1393

AGGAGCTTTGATTCTAATTTTGAAGGAACTGTTG**CAAAAAACA**CAATTCTAGAAGAAGTAAGGAAGGAACACGATCGATTGGGGCAATCTGTGGATGAAGATAAAGCGGTAAGTATTTCAAAGAAAGGATGTAAACAAAATTGA

R S F D S N F E G T V A K N T I L E E V R K E H D R L G Q S V D E D K A V S I S K K G C K Q N *

2 Transcript C2 family — **t-linker**

>Contig_Spider_Gland_98_154

AGGAGCTTTGATTCTAGTCTGGAAGGAACTCTTG**CAAAAACG**CAATTCTAGAAGAAGTGA

R S F D S S L E G T L A K T Q F * K K *

2 Transcript C2 family — **t-linker** — insertion of A ↓ — **PQM-motif** — **h_pep_1c**

>Contig_Spider_Gland_98_953

AGGAGCTTTGATTCTAGTCTGGAAGGAACTCTTG**CAAAAAACG**CAATTCTAGAAGAAGTGAGGAAGGAATACGCTCGATTGGGACAATCTGTGGATGAAGATAAAGCGGTAAAGTATTTCAAAGGAAGGAGTAAACAAAATTGA

R S F D S S L E G T L A K N A I L E E V R K E Y A R L G Q S V D E D K A V K Y F K G R S K Q N *
