# Supplementary material for: Complex precursor structures of cytolytic cupiennins identified in spider venom gland transcriptomes
Source: Sci Rep. 2021 Feb 17;11:4009. doi: 10.1038/s41598-021-83624-z (PMC7889660; doi:10.1038/s41598-021-83624-z)
Supplement: Supplementary file 6 — Supplementary Information 6. [file 41598_2021_83624_MOESM6_ESM.pdf]

**Complex precursor structures of cytolytic cupiennins identified in spider venom gland transcriptomes**

Nature Scientific Reports
Lucia Kuhn-Nentwig
Institute of Ecology and Evolution, University of Bern, Baltzerstrasse 6, 3012 Bern, Switzerland
lucia.kuhn@iee.unibe.ch

# Supporting information S4 Fig.pdf
# Nucleotide sequence analysis of C-terminal Cu 6f and h_pep_2, 3, 4 of transcript family D

Supporting information 4

# Nucleotide sequence analysis of C-terminal Cu 6f and h_pep_2, 3, 4 of transcript family D

**Transcript D family** — **Cupiennin 6f**

```
>Contig_Spider_Gland_98_4    TTCCTTAACCCCTTTAGATGGATGATAAACAAATATCGTGAATGGAAAAACAAAAAAAATTAATAGGAAGATAGGTTGGTTAAATCGTCTCCTGAAATTGCATCTTATGTTTATTAAAAATACGGAAGAAGAAAACAGAAAACAC
                              F  L  N  P  F  R  W  M  I  N  K  Y  R  E  W  K  N  K  K  N  *  *
```

**Transcript D family** — **h_pep_2a** — deletion of A — **h_pep_3a** — **PQM-motif** — **h_pep_4a**

```
>Contig_Spider_Gland_98_336  TTCCTTAACCCCTTTAGATGGATGATAAACAAATATCGTGAATGGAAAAACAAAAAA ATTAATAGGAAGATAGGTTGGTTAAATCGTCTCCTGAAATTGCATCTTATGTTTATTAAAAATACGGAAGAAGAAAACAGAAAACAC
                              F  L  N  P  F  R  W  M  I  N  K  Y  R  E  W  K  N  K  K   I  N  R  K  I  G  W  L  N  R  L  L  K  L  H  L  M  F  I  K  N  T  E  E  E  N  R  K  H
```

dibasic motif (R K) — dibasic motif (R K)

## Hypothetical processing mechanism

**DNA/RNA level**

- **Deletion of A in the nucleotide sequence**

**After translation**

- **Through frameshift identification of new protease cutting motifs: two dibasic motifs as well as a PQM motif**
- **Protelolytic activity results in three peptides**
- **Further C-terminal processing by an unknown ArgC protease**

| | premature peptides | | mature peptides |
|---|---|---|---|
| **h_pep_2a** | :FLNPFRWMINKYREWKNKKIN↓R | **h_pep_2a** | :FLNPFRWMINKYREWKNKKIN |
| **h_pep_3a** | :KIGWLNRLLKLHLMFIKNTEEEN↓R | **h_pep_3a** | :KIGWLNRLLKLHLMFIKNTEEEN |
| | | **h_pep_4a** | :KHSTFLPINFLLTMDKLYMQKIIHALLKNFQCYY |
