# Supplementary material for: Complex precursor structures of cytolytic cupiennins identified in spider venom gland transcriptomes
Source: Sci Rep. 2021 Feb 17;11:4009. doi: 10.1038/s41598-021-83624-z (PMC7889660; doi:10.1038/s41598-021-83624-z)
Supplement: Supplementary file 7 — Supplementary Information 7. [file 41598_2021_83624_MOESM7_ESM.pdf]

Complex precursor structures of cytolytic cupiennins identified in spider venom gland transcriptomes

Nature Scientific Reports
Lucia Kuhn-Nentwig
Institute of Ecology and Evolution, University of Bern, Baltzerstrasse 6, 3012 Bern, Switzerland
lucia.kuhn@iee.unibe.ch

Supporting information S5 Fig.pdf

Graphical overview on transcriptomic data analysis

- Supporting information 5A: workflow for the identification of short linear peptides (LPs)
- Supporting information 5B: overlapping reconstruction of different transcript families (454-seq)
- Supporting information 5C: data evaluation conditions (454-seq)
  Number of identified contigs for transcript family construction (454-seq)
- Supporting information 5D: data evaluation conditions (Illumina3000-Seq)
  Number of identified contigs encoding LPs in transcriptomes of *C. salei* and *C. getazi* (3000 Illumina-Seq)

Supporting information 5A **Transcriptomic data analysis: workflow for the identification of short linear peptides (LPs)**

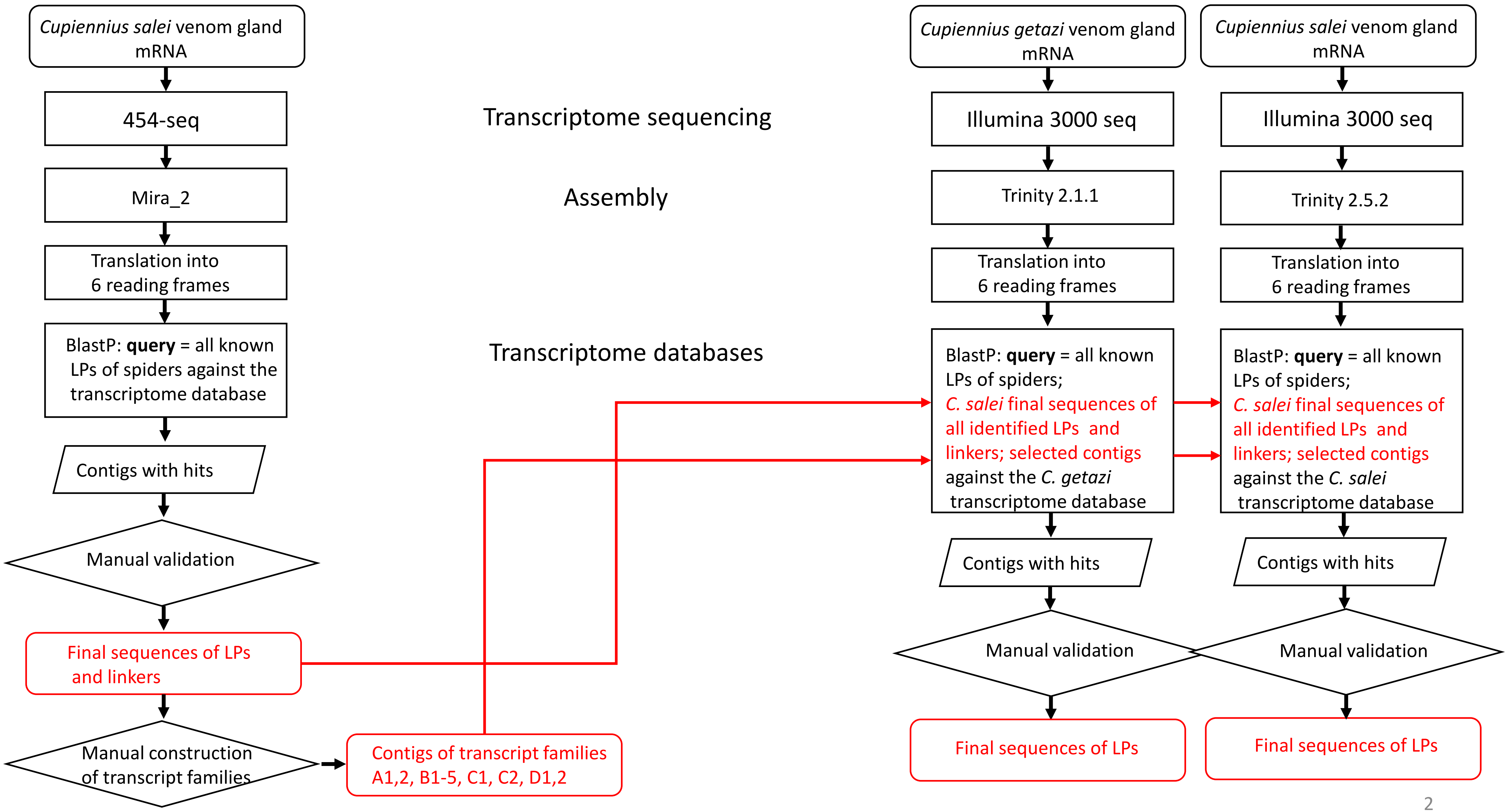

Supporting information 5B

Manual construction of transcript families A1,2, B1-5, C1, C2, D1,2 with contigs obtained by 454 sequencing

1. Identification of contigs containing LPs (e.g., Cu 1a) by BlastP with LPs sequences obtained by proteomics
2. Sorting of all LPs (e.g., Cu 1a) in terms of identical N-terminal and C-terminal linkers
3. Overlapping elongation of sequences by further blasts towards N-terminal direction
4. Overlapping elongation of sequences by further blasts towards C-terminal direction until a clear stop signal is identified

Example transcript family A1 and Cu 1a elongation in direction N-terminus

signal peptide propeptide peptide Cu 1a linker

```
1.                                          ? GFGALFKFLAKKVAKTVAKQAAKQGAKYVVNKQMEG ?
2.                                ? GNEETRSENEIDEEDERGFGALFKFLAKKVAKTVAKQAAKQGAKYVVNKQMEGRTENDSDEEDER  ?
3. MNYLPIICLLLVALASSTTSSSDVPQSELQRKESINSIEDETQRALELTQGNEEETRSENEIDEEDERGFGALFKFLAKK
```

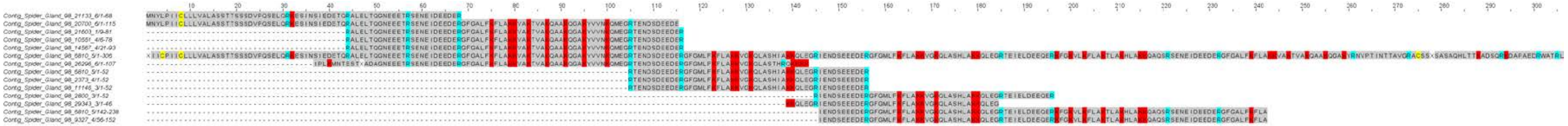

Example transcript family A1 and Cu 1a elongation in direction C-terminus

linker peptide Cu 2a linker peptide Cu 5b linker peptide Cu 1a

```
4. →  -RNENEVEDEDERGFGTILKALAKIAGKVVKKLATKPGATYMLKENLKGRTETEMEVEEERKFGAILKFLAKKLAKHLAKKQAESRTENEVEDEDERGFGALFKFLAKK ?
5.    -----------------------------------------ENLKGRTETEMEVEEERKFGAILKFLAKKLAKHLAKKQAESRTENEVEDEDERGFGALFKFLAKKVAKTVAKQAAKQGAKYVVNKQMEGR
```

linker peptide Cu 5b linker peptide Cu 1a

```
   ENLKGRTETEMEVEEERKFGAILKFLAKKLAKHLAKKQAESRTENEVEDEDERGFGALFKFLAKKVAKTVAKQAAKQGAKYVVNKQMEGR ?
4. →  -------------------------------ESRTENEVEDEDERGFGALFKFLAKKVAKTVAKQAAKQGAKYVVNKQMEGRSLEGTFAKTQFSTOPKKSTOP
5.
```

truncated linker

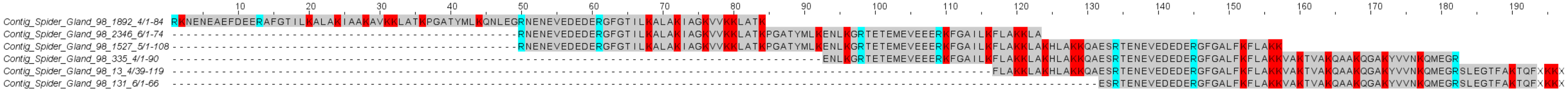

Data evaluation conditions used for

1. Overlapping reconstruction of different transcript families (454-Seq)

Linker / peptide / linker sequence has to be identified in at least 2 contigs
Peptide / linker / peptide sequence has to be identified in at least 2 contigs

```
  linker                     peptide                        linker
**RSEN**EIDE**EDER**GFGALFKFLAKKVAKTVAKQAAKQGAKYVVNKQME**GRTEN**DSDE**EDER**
```

linker — peptide — linker

RSENEIDE**EDER**GFGALFKFLAKKVAKTVAKQAAKQGAKYVVNKQME**GRTEN**DSDE**EDER**

peptide — linker — peptide

KFGAILKFLAKKLAKHLAKKQAES**RTEN**EVED**EDER**GFGALFKFLAKKVAKTVAKQAAKQGAKYVVNKQME**G**

iPQM-motif → RTEN; **PQM**-motif → EDER

glycine for C-terminal amidation of the peptide

2. Number [N] of identified contigs for transcript family construction (454-seq)

Transcript family	A1,2	B1-5	C1	C2	D1, 2
N	654	483	851	90	489

Supporting information 5D

1. Identification of LPs in transcriptomes of *C. salei* and *C. getazi* (3000 Illumina-Seq)

BlastP searches (E-threshold E 0.0001) were performed with all identified LPs of *C. salei* obtained by 454-Seq and Edman Seq (UniProtKB)
with selected contigs identified for the construction of the transcript families A1,2, B1-5, C1, C2, D1,2
with all LPs sequences (peptide sequences from UniProtKB and translated mRNA sequences from ENA)
available for oxyopids, lycosids, zodariids as **query**
and the 6 frame translated contigs (3000 Illumina-Seq) of *C. salei* and *C.getazi* as **databases**

2. Sequence conditions for the selection of new LPs identified in the above mentioned transcriptomes

Linker / peptide / linker sequence has to be identified in 1 contig

```
 linker                  peptide                     linker
RSENEIDEEDERGFGALFKFLAKKVAKTVAKQAAKQGAKYVVNKQMEGRTENDSDEEDER
```

Linker / peptide / linker sequence has to be identified at least in 2 contigs, and exhibiting at least 12 bps (iPQM-motif and PQM-motif) if the linkers are truncated

```
truncated linker          peptide           truncated linker
          EDERGFGALFKFLAKKVAKTVAKQAAKQGAKYVVNKQMEGRTEN
```

12 bps long
PQM motif

12 bps long
iPQM motif

3. Number of identified contigs encoding LPs in

C. salei (Illumina-3000-Seq) transcriptome: N = 301
C. getazi (Illumina-3000-Seq) transcriptome: N = 158
